# Deciphering tissue‐based proteome signatures revealed novel subtyping and prognostic markers for thymic epithelial tumors

**DOI:** 10.1002/1878-0261.12642

**Published:** 2020-02-06

**Authors:** Xin Ku, Qiangling Sun, Lei Zhu, Zhitao Gu, Yuchen Han, Ning Xu, Chen Meng, Xiaohua Yang, Wei Yan, Wentao Fang

**Affiliations:** ^1^ Shanghai Center for Systems Biomedicine Key Laboratory of Systems Biomedicine (Ministry of Education) Shanghai Jiao Tong University China; ^2^ Department of Thoracic Surgery Shanghai Chest Hospital Shanghai Jiao Tong University China; ^3^ Thoracic Cancer Institute Shanghai Chest Hospital Shanghai Jiao Tong University China; ^4^ Department of Pathology Shanghai Chest Hospital Shanghai Jiao Tong University China; ^5^ Bavarian Center for Biomolecular Mass Spectrometry Technical University of Munich Freising Germany; ^6^ Central Lab Shanghai Chest Hospital Shanghai Jiao Tong University China

**Keywords:** molecular profiling, protein classifiers, targeted proteomics, thymic epithelial tumors, WHO classification

## Abstract

Thymic epithelial tumors (TETs) belong to a group of tumors that rarely occur, but have unresolved mechanisms and heterogeneous clinical behaviors. Current care of TET patients demands biomarkers of high sensitivity and specificity for accurate histological classification and prognosis management. In this study, 134 fresh‐frozen tissue samples (84 tumor, 40 tumor adjacent, and 10 normal thymus) were recruited to generate a quantitative and systematic view of proteomic landscape of TETs. Among them, 90 samples were analyzed by data‐independent acquisition mass spectrometry (DIA‐MS) leading to discovery of novel classifying molecules among different TET subtypes. The correlation between clinical outcome and the identified molecules was probed, and the prioritized proteins of interest were further validated on the remaining samples (*n* = 44) via parallel reaction monitoring (PRM) as well as immunohistochemical and confocal imaging analysis. In particular, two proteins, the cellular mRNA deadenylase CCR4 (carbon catabolite repressor 4)‐NOT (negative on TATA) complex subunit 2/9 (CNOT2/9) and the serine hydroxymethyltransferase that catalyzes the reversible interconversions of serine and glycine (SHMT1), were found at dramatic low levels in the thymic epithelia of more malignant subtype, thymic squamous cell carcinoma (TSCC). Interestingly, the mRNA levels of these two genes were shown to be closely correlated with prognosis of the TET patients. These results extended the existing human tissue proteome atlas and allowed us to identify several new protein classifiers for TET subtyping. Newly identified subtyping and prognosis markers, CNOT2/9 and SHMT1, will expand current diagnostic arsenal in terms of higher specificity and prognostic insights for TET diagnosis and management.

## Graphical abstract

Thymic epithelial tumors (TETs) belong to a group of tumors that rarely occur, but have unresolved mechanisms and heterogeneous clinical behaviors. Current care of TET patients demands protein biomarkers of high sensitivity and specificity for accurate histological classification. In this study, we surveyed 134 tissue proteomes, identified, and validated a suite of protein classifiers associated with different TET subtypes.

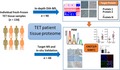

**Abbreviations**
CNOT2/9: Carbon catabolite repressor 4‐negative on TATA complex subunit 2/9
DDA: Data‐dependent acquisition
DIA: Data‐independent acquisition
FFPE: Formalin‐fixed paraffin‐embedded
HE: Hematoxylin and eosin
IHC: Immunohistochemistry
PCA: Principal component analysis
PLS‐DA: Partial least squares discriminant analysis
PRM: Parallel reaction monitoring
SHMT1: Serine hydroxymethyltransferase
TC: Thymic carcinoma
TdT: DNA nucleotidylexotransferase
TET: Thymic epithelial tumors
TSCC: Thymic squamous cell carcinoma

## 1 Introduction

Located in the anterior mediastinum, thymus plays a crucial role in the maturation and release of T lymphocytes into the circulation in adaptive immunity (Blackburn and Manley, 2004; Boehm and Swann, 2013; Hogquist *et al.*, 2005; Takahama *et al.*, 2017). Alterations of its controlled activity have been shown to result in a wide spectrum of clinical behaviors including many autoimmune disorders (Bernard *et al.*, 2016; Josefowicz *et al.*, 2012; Sakaguchi *et al.*, 2008; Savage *et al.*, 2014) and thymic epithelial tumors (TETs). Among them, the TETs, mainly including thymoma and thymic carcinoma (TC), are the most common tumors of the anterior mediastinum (20–50% in adults) with a notable proportion present at an advantage stage (Engels and Pfeiffer, 2003; Thomas *et al.*, 2015). The 5‐year median survival rate of TETs is 69% in thymomas and 19% in TC (Hamaji and Burt, 2015; Scorsetti *et al.*, 2016). Although with relatively rare overall occurrence (approximately three cases per million people per year worldwide (Engels, 2010)), the TETs have gained specific attentions clinically because the TETs are considered to originate from thymic epithelial cells which can affect many important features of non‐neoplastic lymphocytes (Scorsetti *et al.*, 2016; Venuta *et al.*, 2012). On the other hand, increased risk of extrathymic secondary cancers among TET patients has been reported (Filosso *et al.*, 2013; Kamata *et al.*, 2017; Kumar *et al.*, 2018; Pan *et al.*, 2001; Travis *et al.*, 2003; Weksler *et al.*, 2012).

Due to the extreme heterogeneity on both morphology and clinical behaviors, TETs have been lacking commonly accepted subtyping system that further limits their diagnosis precision. Over the years, more than 10 subclassification systems have been introduced (Bernatz *et al.*, 1973; den Bakker et al., 2014; Koppitz *et al.*, 2012; Lattes and Pachter, 1962; Levine and Rosai, 1978; Muller‐Hermelink and Marx, 2000; Suster and Moran, 1999a; Suster and Moran, 1999b; Suster and Moran, 1999c). Among them, the 2014 World Health Organization (WHO) TET classification is the most adopted system by many pathologists (Marx *et al.*, 2014), in which the TETs are mainly subdivided into A, AB, B1, B2, and B3 for thymoma and TC. The type A thymoma derives from the central medulla region of thymus and has neoplastic cells with spindle or oval shape, whereas the type B thymomas originate from thymic cortex on the periphery with the presence of immature lymphocytes (Weksler and Lu, 2014). Type AB thymoma is generally regarded to combine features from both type A and type B (Green *et al.*, 2015; Khaki *et al.*, 2013). The TC, often occurring in 5–10% of the TET patients (Kelly, 2013; Lamarca *et al.*, 2013; Ried *et al.*, 2016), consists of a variety of diverse tumors, among which thymic squamous cell carcinoma (TSCC) is the most frequently observed (Marx *et al.*, 2014; Moser *et al.*, 2014; Scorsetti *et al.*, 2016; Takahama *et al.*, 2017; Weissferdt *et al.*, 2012; Wu *et al.*, 2016). Such WHO classification of TET is mainly based on histological characterization. Immunohistochemistry (IHC) assays on several TET histology markers such as P63, EMA (epithelial membrane antigen), and CD5 have been developed and clinically practiced (Carter *et al.*, 2017; Marx *et al.*, 2014; Moran and Suster, 2008; Roden *et al.*, 2015; Weissferdt *et al.*, 2012). However, these existing classifying markers have limited capability in differentiating complicated cases (e.g., types A, B3, and TC) due to inadequate specificity on such highly heterogeneous disease phenotypes.

Therefore, it has been highly demanded to identify key signature molecules that can not only facilitate accurate subtyping of TETs, but also provide molecular insights into the unknown etiology of these rare, heterogeneous, but mechanistically interesting diseases. The Cancer Genome Atlas (TCGA) research network and other scientists have initiated such attacks by systematic genomic studies on TETs, aiming for uncovering the genetic landscape of these rare tumors (Lee *et al.*, 2017; Radovich *et al.*, 2018). At the proteomic domain, two pioneered studies have recently been reported to identify marker proteins to differentiate TET subtypes for diagnostic purpose (Wang *et al.*, 2016; Zhao *et al.*, 2018). Due to the limited tissue sample resource for these rare diseases, these studies had to work on the less proteomic‐friendly formalin‐fixed paraffin‐embedded (FFPE) tissue samples at a limited scale and even with a pooled sample approach (Zhao *et al.*, 2018). However, it would be ideal to conduct clinical proteomic analysis on relatively large‐scale samples from individual patients that is rich in clinical information and with more statistical power rather than a stand‐alone assay with pooled sample profiles. In this study, we started to practice such clinical proteomic platform by analyzing the proteomic‐friendly fresh‐frozen tissue samples consisting of the subtypes A, AB, B1, B2, B3, and TC and normal thymus. We specifically select the recently developed data‐independent acquisition mass spectrometry (DIA‐MS) methodology for discovery studies, which has shown great value in analyzing limited and nonrenewable clinical specimen (Gillet *et al.*, 2012; Meyer and Schilling, 2017). By generating a permanent ‘digital map’ of each analyzed sample proteome with high reproducibility, the acquired data can be analyzed, re‐analyzed, and mined *in silico* to identify and quantify thousands of proteins across multiple samples (Collins *et al.*, 2017; Gillet *et al.*, 2016; Guo *et al.*, 2015). Parallel reaction monitoring (PRM) technique was chosen to validate the protein classifiers identified from DIA‐MS studies. By acquiring full MS2 product ion spectra of selected precursors, PRM monitored simultaneously all detectable product ions at high resolution and accuracy, without preselection of target transitions prior to MS analysis. Recently, PRM has demonstrated clear advantages over the conventional ‘golden standard’, selected reaction monitoring (SRM) approach, for analyzing biomarkers in complex samples such as tissues and body fluids (Gallien *et al.*, 2012; Kim *et al.*, 2015; Peterson *et al.*, 2012; Ronsein *et al.*, 2015). PRM technique offers higher peptide identification specificity and quantitative accuracy by high‐resolution MS acquisition of all potential product ions of targeted peptide as compared with only 3–5 preselected product ions (transitions) applied in SRM. Meanwhile, without preselection of transitions, PRM has diminished substantially the effort to develop SRM assays. Altogether, the generated clinical TET proteome profiling data resource can be used in many ways to explore protein expression and its regulation in thymus. With more upcoming proteomic data acquired from patient samples in the future, we will be able to conduct such big data analysis eventually, to deepen our insight into the molecular fingerprints of TETs and guiding the therapeutic and prognostic management in a precision manner.

## 2 Materials and methods

### 2.1 Patient cohort

This study was approved by the Ethical Committee of Shanghai Chest Hospital, following the ethical guidelines and standards set by the Declaration of Helsinki. Informed consent forms were received from all patients included in this study. A total number of 134 fresh‐frozen tissue samples were obtained from the Tissue Biobank of Shanghai Chest Hospital, encompassing thymoma/TC tissues and the corresponding pairwise tumor‐adjacent tissues as well as tissues from normal thymus. The disease tissues are of all major subtypes (A, AB, B1, B2, B3, and TSCC) from TET patients undergoing surgery, and 10 tissues from normal thymus were obtained from patients undergoing corrective cardiovascular surgery. Most tissue samples recruited for the analysis were naive samples (without pre‐radio‐ or chemotherapy, detailed patient information including Masaoka‐Koga stages and malignant cell percentages, see Table S1). Adjacent biopsies were dissected approximately 2 cm from the tumor. The histological subtype of each recruited sample was evaluated by two pathologists according to the WHO criteria using hematoxylin and eosin (HE)‐stained sections. For confusing cases and disagreement, a third pathologist was included for further discussion.

### 2.2 Tissue homogenization and protein extraction

After careful review of all the histological subtypes, lysis buffer was added into each sample, in which the corresponding tissue block was precut into small pieces (~ 1 mm^3^). The lysis buffer contains 0.2% homemade acid‐labile surfactant (ALS) (Ross *et al.*, 2002) in 20 mm HEPES buffer with 1X protease inhibitor (Roche, Basel, Switzerland) and was optimized specially for thymus tissue protein extraction in our previous study, resulting in a high recovery of versatile proteins (Sun *et al.*, 2018). All the samples were placed individually in homogenization tube with precooled ceramic beads at 4 °C. After homogenization, the samples were kept half an hour on ice. Then, the lysed cells were centrifuged at 20 000 ***g*** force for 0.5 h at 4 °C. Standard BCA assay was applied to detect protein concentrations of all samples.

### 2.3 Protein digestion and peptide purification

After lysis, the proteins were denatured by 6 m urea at room temperature for 1 h. Then, tris(2‐carboxyethyl)phosphine (5 mm) was added to reduce the proteins at room temperature for half an hour. To alkylate the reduced proteins, iodoacetamide (IAA) was applied in each sample in 6.25 mm. The reaction mixture was incubated for 0.5 h at RT in dark place. After that, each sample was diluted with six volumes of HEPES buffer (50 mm, pH = 8.2) to ensure that urea concentration is below 1 m. Sequence‐modified trypsin [Promega, Madison, WI, USA 1 : 100 (w/w)] was added to each sample and incubated on an end‐over‐end shaker for 12 h at 37 °C. After digestion, the peptide mixture was quenched and acidified by phosphoric acid to pH = 2. Then, the acidic peptide mixture was loaded onto a pre‐activated C‐18 cartridge (96‐well plate; Thermo Fisher, Waltham, MA, USA). Desalting was conducted by washing three times with 0.1% formic acid (200 µL). After that, peptides were eluted with 50% ACN and dried under vacuum with a SpeedVac.

### 2.4 Liquid chromatography–Tandem mass spectrometry (LC‐MS/MS) analysis

Before subjected to mass spectrometric analysis, each peptide sample was dissolved in 0.1% FA (formic acid) to 0.5 mg·mL^−1^ and iRT Kit (Biognosys, Zurich, Switzerland) was added (according to manufacturer’s instruction). A nanoflow LC (Dionex UltiMate 3000; Thermo Fisher Scientific) was coupled to ultra‐high‐resolution mass spectrometer (Orbitrap Fusion; Thermo Fisher Scientific). For proteomic analysis, 1 μg peptide (2 μL) was separated by a self‐packed analytical column (3 μm particle, 75 μm × 150 mm, Inspire C18; Dikma, Markham, Canada) at 300 nL·min^−1^. Binary elution buffer system containing buffer A (0.1% FA in ddH_2_O) and buffer B (0.1% formic acid in ACN) was used to analyze peptides in a 62‐min elution time using 7% to 35% of buffer B. For spectral library generation, the high‐resolution mass spectrometer (Orbitrap Fusion) worked in data‐dependent acquisition (DDA) mode. Full scan (MS1, mass range: 350–1550*m/z*) was obtained at 60 000 resolution (at *m/z* 400) with an automatic gain control of 200 000 for a maximum collection time of 100 ms. For MS2 acquisition, the mass resolution was tuned to 30 000 and spectra were recorded in top speed mode (maximum 3 s). Fragments were generated by HCD (higher energy collision‐induced dissociation, 30% normalized collision energy) and recorded when accumulated at a target value of 10 000 or max 35‐ms injection time). Recurrence of precursors was not considered within 60 s (dynamic exclusion).

DIA analysis was carried out using the same LC system and mass spectrometer as they were for DDA analysis. The same flow rate, same gradient, analytical column, and same buffers were applied. Orbitrap Fusion was operated in *t*‐MS^2^ mode. The duty cycle started from a high‐resolution MS1 scan event acquired with 60 000 resolution and followed by 30 DIA sequential scans, each with an isolation windows of 21 *m/z* (1 *m/z* overlapped between each window) at 30 000 resolution to cover the precursor mass range from *m/z* 400 to 1000. The rest of the acquisition parameters remained identical to the DDA analyses. All these generated raw MS files were deposited to the PRIDE repository with the dataset identifier PXD016498.

### 2.5 Spectral library construction and data analysis

The spectral library was built up by combination of 14 DDA files (acquired from different tissue subtypes). The peak lists were directly picked from acquired raw MS files and were further used to search against UniProt Protein Database (Homo sapiens, 2016.09.16, supplemented with Biognosys iRT peptides fasta file which is available on Biognosys website) by SEQUEST implemented in Proteome Discoverer (version 1.4; Thermo Fisher Scientific). Spectral matching was conducted using oxidation on methionine as dynamic modification, and carbamidomethylation on cysteine residues as static modification. Up to two missed cleavages were tolerated while trypsin was specified as proteolytic digesting enzyme. For precursors, the mass tolerance was allowed for 10 ppm, while for fragments, the mass tolerance was restricted to 0.02 Da. Identified peptides were filtered in Proteome Discoverer at high confidence level. Target‐decoy search strategy was applied to estimate protein false discovery rate (FDR), which was filtered at 1%. The spectral library was generated by combining the spectra‐match information from all DDA data using Spectronaut (version 9.0, Biognosys) at a FDR of 0.01 following the standard format for custom libraries in Spectronaut (Manual for Spectronaut, available on Biognosys website) (Bruderer *et al.*, 2015; Bruderer *et al.*, 2016). It contained 3737 protein groups, 29 382 peptides, and 233 397 fragments. All DIA raw data files were imported in Spectronaut, after being converted in HTRMS Converter (Biognosys). A dynamic window for the XIC extraction and a nonlinear iRT calibration strategy were applied. Decoy generation was set to scramble (no decoy limit). The identification was performed by using the normal distribution estimator, including MS1 scoring and the dynamic score refinement (Bruderer *et al.*, 2017). By summing the peak areas of all their respective fragment ions for MS2, peptide intensity was calculated. Protein intensities were calculated by summing the intensities of all their respective peptides (see Table S2 for the data matrix of all raw protein abundances) in each sample and were log_2_‐transformed (Bruderer *et al.*, 2015; Bruderer *et al.*, 2016; Bruderer *et al.*, 2017). A cross‐run normalization was performed by using the total peak area as normalization base. No preselection or filtering on either peptide or protein level was applied. Significance analysis of protein abundance variations was carried out using two‐sided Student’s t‐test, and all reported *P*‐values were adjusted according to Benjamini and Hochberg correction when doing multiple comparisons. Further, data interpretation and functional annotation were performed using DAVID (v6.8) and ingenuity pathway analysis (Qiagen, v01‐12). Heat maps and PCA graphs were plotted using R (v 3.3.2) with the packages pheatmap and ggplot installed.

### 2.6 Parallel reaction monitoring and data analysis

From PRM analysis, fully tryptic precursors of target proteins were selected (see Table S3 for precursor information, such as *m/z*, charge, and retention time). LC‐MS/MS was performed using the same LC and MS instruments as applied in DIA‐MS. For each analysis, 1 μg dissolved peptides (in 2 μL buffer A) was delivered to the analytical column (3 μm, 150 mm × 75 μm, self‐packed, Inspire C18, Dikma, Canada) and separated using the same gradient and flow rate as in DIA analysis. In MS1, the precursor scan was acquired at 60 000 resolution, with the AGC set to 4e5 and 100‐ms maximum injection time. The MS2 spectra were acquired 30 000 resolution, with the AGC set to 5e4, isolation width of 1.5 *m/z*, and 54‐ms maximum injection time.

Raw PRM data were imported into skyline (v4.2, https://www.skyline.ms). The precursor charges were restricted to 2, 3, or 4, and the ion charge was set as single with a y or b ion type. The MS2 matching tolerance was set at 0.05 *m/z*. Interfering transitions were manually removed based on visual inspection. The protein quantification was obtained by adding up all peptide intensities of the corresponding protein.

### 2.7 Immunohistochemistry staining and analysis

Immunostainings were performed with various primary antibodies (see Table S4 for more information about antibodies used in the study) and secondary antibodies of the Dako REAL EnVision Detection System (Dako, Glostrup, Denmark). Paraffin‐embedded specimens were sectioned at 4 μm thickness and baked at 65 °C for 2 h, and then, xylene was used to deparaffinize the tissue sections followed by washing with gradient alcohol. Tissue sections were then rehydrated with water and pretreated for antigen retrieval in a microwave oven at 95 °C for 25 min in citrate‐EDTA buffer. Then, 3% H_2_O_2_ solution was added to block endogenous peroxidase activity. After three times of wash with PBS, the sample was blocked with goat serum for 10 min at 37 °C and then incubated with primary antibody at 4 °C overnight. Then, the sample was further washed three times using PBS and incubated with secondary antibody at RT for 1 h. The immunoreactivity was visualized using 3,30‐diaminobenzidine chromogen for 5 min. The whole slide images were acquired by digital microscopy scanner Pannoramic MIDI (3DHISTECH, Ltd., Budapest, Hungary). Semiquantitative analysis of IHC images was calculated manually with the help of CaseViewer 2.3 (3DHISTECH, Ltd.), by multiplying the scores obtained both on staining intensity and on proportion of positive cells of the images (Du *et al.*, 2016).

### 2.8 Immunofluorescent assay

The fresh thymoma tissues were placed in 4% paraformaldehyde for 24 h and then transferred to 20% sucrose solution. After 48‐h fixation, the fixative was replaced with 30% sucrose solution for further 72 h. Then, the specimens were put on Tissue‐Tek (Specimen Holder, OCT Polysciences) and were rapidly frozen on dry ice. After that, tissues were cut into sections at a thickness of 4 μm using a Leica Cryostat Microtome (CM1850, Leica Biosystem, Nussloch, Deutschland). For staining, sections were incubated on slides with the mixed antibodies of SHMT1/CK19 or CNOT2/CK19 (1 : 100) in a humid atmosphere at 4 °C overnight. After being rinsed by PBS for three times, the sections were kept at RT with a donkey‐derived secondary antibody (conjugated to Alexa Fluor 488 and 594; Jackson Immunoresearch, West Grove, PA, USA) for 1 h). Nuclear dye 4′,6‐diamidino‐2‐phenylindole (DAPI) was used to counterstain the sections for 30 s. After being washed by PBS, the sections were mounted with Dako fluorescence mounting medium. The super‐resolution imaging was investigated by an inverted Zeiss LSM710 microscope (Oberkochen, Germany). The laser wavelengths for excitation were 405 nm (DAPI), 488 nm (Alexa 488), and 594 nm (Alexa 594). Emission was collected sequentially through bandpass filters: 420–480 nm for DAPI, 495–575 nm for Alexa 488, and 570–650 nm for Alexa 594 (see Table S4 for more information about antibodies used in the study).

## 3 Results

### 3.1 Overview of patient demographics and their proteomic profiles

A total number of 134 samples (90 for discovery and 44 for validation) were recruited in this study. The clinical and pathological characteristics of these patient samples are summarized in Table 1 (see Table S1 for more details). The included patients represented all major WHO histological subtypes at different pathological stages according to the Masaoka‐Koga system. The stages of most TSCC patients were Masaoka Stage II or higher, while for thymoma, patients covered all stages (I–IV).

**Table 1:** Clinical characteristics of patient samples included in this study.

	Subtype	Sample cohort	Masaoka Stage	Age (mean ± SD)	Gender (male/female)
Discovery cohort					
Thymoma	Type A	5	Stage I: 12 Stage II: 11 Stage III: 4 Stage IV: 3	53 ± 11	13/17
	Type AB	8			
	Type B1	4			
	Type B2	8			
	Type B3	5			
TSCC		10	Stage I: 0 Stage II: 2 Stage III: 6 Stage IV: 2	65 ± 13	9/1
Tumor adjacent		40	–	56 ± 13	22/18
Normal thymus		10	–	52 ± 13	7/3
Validation cohort					
Thymoma	Type A	7	Stage I: 2 Stage II: 12 Stage III: 4 Stage IV: 9	50 ± 15	16/11
	Type B1	7			
	Type B2	6			
	Type B3	7			
TSCC		17	Stage I: 0 Stage II: 5 Stage III: 6 Stage IV: 6	58 ± 6	9/8

**Figure 1:**
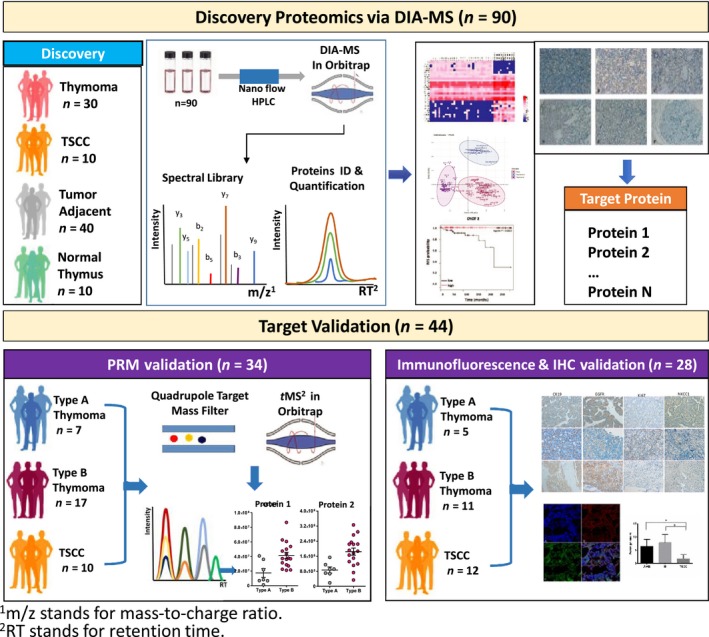
Workflow of mass‐spectrometry‐based proteomic analysis of TETs. The study was carried out with two independent patient sample batches, namely discovery (*n* = 90) and validation (*n* = 44) batch. For both batches, individual specimen was processed following typical proteomic sample preparation protocol. For discovery study, samples were analyzed by DIA. A quantitative ‘proteome map’ was thus generated for each patient sample, of which proteins of interest were prioritized using various bioinformatic tools. For validation purpose, 34 samples were analyzed by PRM and 28 for IHC as well as immunofluorescence stainings (18 samples were validated by both PRM and IHC).

The workflow is summarized in Fig. 1. A typical proteomic sample preparation procedure was applied for all samples (see Materials and methods section). For discovery proteomics, 90 specimen including 30 thymomas (5 type A, 8 type AB, 4 type B1, 8 type B2, and 5 type B3), 10 TSCC samples with their corresponding tumor‐adjacent samples (*n* = 40), and 10 normal thymus tissue samples were analyzed by LC‐MS/MS to generate quantitative ‘proteome map’ for each patient sample, of which proteins of interest were prioritized. For validation purpose, 44 tissues were further incorporated, with 34 applied in PRM and 28 applied in IHC/immunofluorescence stainings (18 samples were validated by both PRM and IHC).

**Figure 2:**
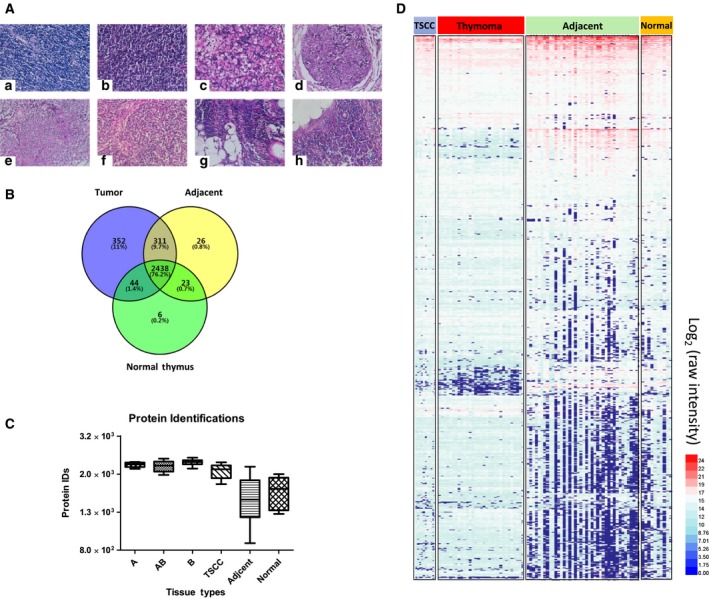
Histological and proteomic characteristics of different thymic tissues. (A) Selected HE‐stained TET tissue sections of different WHO subtypes (at 400× magnification). a) B1; b) B2; c) B3; d) TSCC; e) A; f) AB; g) tumor adjacent; and h) normal thymus. (B) Venn diagram showing the overlaps of proteomic profiles among tumor tissues, adjacent tissues, and normal thymus. (C) Protein identifications from different tissues (type A, *n* = 5; type AB, *n* = 8; type B, *n* = 17; type TSCC, *n* = 10; type adjacent, *n* = 40; type normal, *n* = 10), and data were shown as mean ± SEM. (D) Heat map analysis of individual proteomic profile of all samples, revealing significant variations of protein expression profiles across different tissue subtypes.

In this study, we carefully evaluated tissue subtypes of all samples via HE‐staining‐based pathological review (see Fig. 2A for representative images). Thymoma was featured by observed organ‐like characteristics, such as medullary differentiation region and perivascular spaces (PVS) (see Fig. S1 for examples of such typical structures at 200× magnification). In type B thymoma, different levels of immature T lymphocyte infiltration were observed (T lymphocyte density decreases from B1 to B3, images a to c). Type A thymoma was composed of tumor cells in spindle/oval shape, with few or no admixed immature lymphocytes (image e). Unlike thymomas, TSCC showed lack of resemblance to the normal thymic cytoarchitecture (image d), such as PVS, medullary differentiation regions, and admixed immature lymphocytes. Interestingly, adipose components were found rich in tumor‐adjacent (image g) and normal thymus tissues (image h).

Discovery proteomics allowed a total of 3200+ protein groups quantified from 90 DIA‐MS runs. In tumor samples, we identified more than 3000 proteins, while in adjacent and normal tissues, about 2500–2800 proteins were identified (Fig. 2B). Protein identifications in tumor‐adjacent (adjacent) and normal thymus (normal) samples were remarkably lower than that in tumor tissues (Fig. 2C). Overall evaluation of these quantified proteomes by clustering analysis reveals similar protein expression patterns between adjacent tissue and normal thymus, which is significantly different from that of thymoma and TSCC samples, possibly due to their adipose‐dominating feature of degenerated thymus tissue in aged adults (Fig. 2D). Similar results could also be obtained by principal component analysis (PCA) on these quantified proteomes (Figs S2 and S3).

Proteome map from normal thymus tissue was of great interest but not well characterized in comparison with that from other tissues, possibly due to the limitation of sample resource. Previous proteomic analyses reported a list of around 1200 proteins identified in FFPE samples (Wang *et al.*, 2016). In this study, we took advantages of the fresh‐frozen tissue samples and an in‐house improved efficient protein extraction protocol, and identified more than 2500 proteins from the normal thymus tissues (Sun *et al.*, 2018). This information will further extend the existing normal human tissue proteome atlas (Wang *et al.*, 2019), providing a comprehensive baseline map of protein expression in thymus.

### 3.2 Molecular signatures of type A and type B thymoma

**Figure 3:**
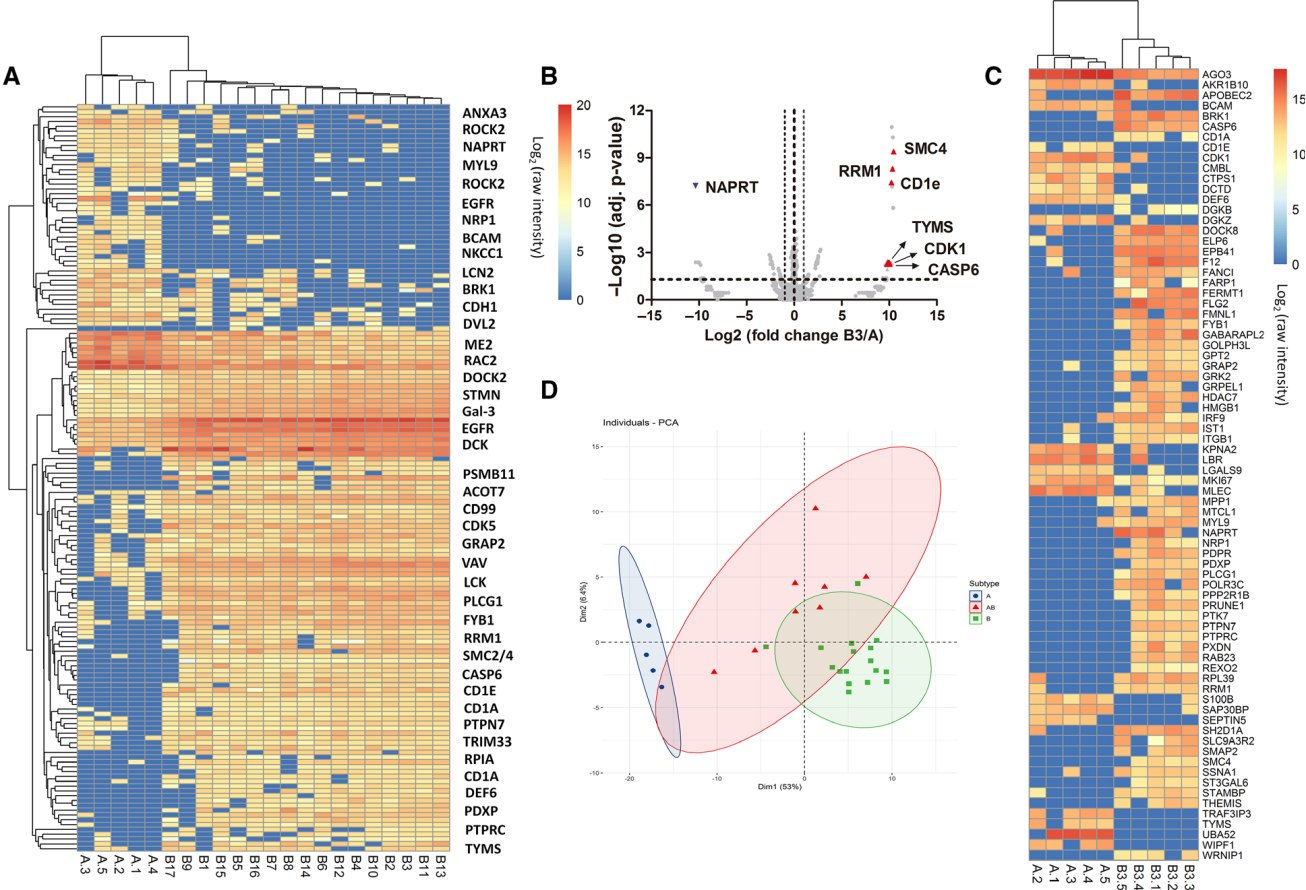
Molecular signatures of type A and type B thymoma. (A) Heat map illustration of 155 differentially expressed proteins between type A (*n* = 5) and type B (*n* = 17) thymoma, and representative proteins were labeled on the right of each featured cluster (for a full list of all proteins on the figure, see Table S5). (B) Increased expression of proteins such as RRM1, SMC4, TYMS, CDK, and CASP6 was found in the B3 proteome shown in volcano plot B3 (*n* = 5) and A (*n* = 5). (C) Further prioritized proteins by PLS‐DA were presented by heat map analysis with hierarchical clustering. (D) PCA plot showed poor discrimination of type AB from type A and type B using the 155 differential proteins between type A (*n* = 5) and type B (*n* = 17). Statistical significances were determined by Student’s *t*‐tests (*P* < 0.05).

In thymoma, some physiological functions of normal thymus were retained that involve in differentiation and homing of the lymphocytes. This feature of thymoma was reflexed by its epithelial components, medullary and cortical cells, shown in the two significantly different subtypes of thymomas [type A and type B (Strobel *et al.*, 2010)]. In this study, we specifically compared proteomic profiles of type A and type B thymomas. Among the 3100 proteins identified from thymoma samples of subtypes A, AB, and B (Fig. S4A), 155 proteins were differentially expressed between type A and type B, not between Adjacent A and Adjacent B tissues (representative proteins were labeled on the right of each featured cluster, *P* < 0.05, Fig. 3A, Table S5). The main biological functions of these proteins identified by ingenuity pathway analysis (IPA) were cell movement, leukopoiesis, and lymphocyte quantity (Fig. S4B). Further network analysis revealed that proteins overexpressed in type B thymomas, such as PTPRC, LCK, PTPN7, GRAP2, PLCG1, VAV1, and TYMS, were highly relevant to T‐cell receptor signaling, T helper cell signaling, cytotoxic T lymphocyte, and phagocytosis signaling pathways and played pivotal roles in proliferation and differentiation of lymphocytes. On the other hand, proteins overexpressed in type A thymomas, such as MYL9, ROCK2, CDH1, RRAS2, GNG12, EGFR, ITGB1, BRK1, SEPT5, and NRP1, were mapped to canonical pathways including IL‐8, Rho family GTPase, and epithelial adherens junction signaling. The upstream regulator analysis showed these differential proteins with their overexpression in type A thymoma were likely regulated by HBEGF, LEF1, and GLIPR1.

Precise classification between types A and B3 was of great clinical importance since type A and type B3 thymoma represent the two opposite extremes on prognosis, with 10‐year survival at nearly 100% and 30–40% for type A and type B3, respectively. Current clinical subtyping of these two thymomas heavily relied on cell morphology that was subject to pathologist’s observation and experience. Many existing histological markers such as cytokeratin (panCK) and DNA nucleotidylexotransferase (TdT) showed similar immunohistochemical staining patterns between type A and type B3, and there are so far no specific molecular markers to assist clinical diagnosis and clear subtyping for these two types of tumor. After mining the proteome map of these two sample groups using Student’s *t*‐test, more than 100 proteins were prioritized as differentially expressed proteins between type A and type B3. Increased expression of proteins such as RRM1, SMC4, TYMS, CDK, and CASP6 was found in the B3 proteome (Fig. 3B). Using partial least squares discrimination analysis (PLS‐DA), we further prioritized 76 protein molecules to discriminate type A and type B3 (*Q* ^2^> 0.5, Fig. S5). Hierarchical clustering showed that using these selected protein classifiers, the samples could be clearly clustered into two groups that were in line with their histological classifications (Fig. 3C).

Type AB thymoma by definition is a thymic epithelial neoplasm composed of both features from lymphocyte‐poor type A‐like components and lymphocyte‐rich type B‐like components. Therefore, clinical subtyping of type AB thymoma encountered lots of discrepancies for complex samples (Fig. S6). The proteomic profile of type AB combines the features from both type A and type B, leading to poor discrimination of type AB from type A and type B using the 155 differential proteins between type A and type B (see PCA results in Fig. 3D).

### 3.3 Evaluation of existing histological classifiers of type A and type B thymoma

**Figure 4:**
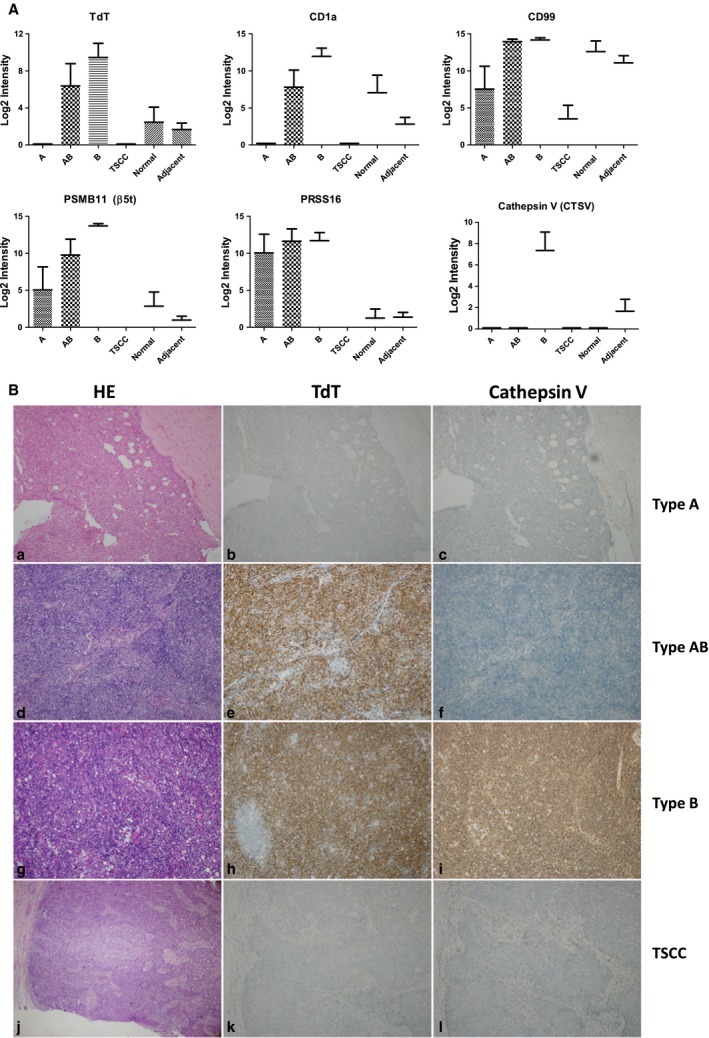
Evaluation of existing histological markers to differentiate TET subtypes. (A) Marker protein abundances across different tissue types quantified from MS data (type A, *n* = 5; type AB, *n* = 8; type B, *n* = 17; type TSCC, *n* = 10; type adjacent, *n* = 40; type normal, *n* = 10), and data were shown as mean ± SEM. (B) HE and IHC staining of two clinical IHC markers, TdT and Cathepsin V, across different TET subtypes. IHC images were at 100× magnification. Results showed Cathepsin V is highly specific to type B thymoma, whereas TdT showed less specific than Cathepsin V, with expression also found in type AB.

Recently, WHO had suggested a dozen of histochemical markers to differentiate type A and type B thymoma, including TdT, CD1a, CD99, PSMB11 (Beta5T), PRSS16, Cathepsin V, and desmin (Marx *et al.*, 2014). However, a systematic overview of the expression patterns for all these markers across all TET subtypes was not achieved previously due to intrinsic low throughput and complicated experimental procedures of clinical IHC assays. In this study, we looked into protein expression spectrum of these WHO histochemical markers in our quantified proteome map. As shown in Fig. 4A, TdT and CD1a appeared to be very similar, with both the highest expressions in type B, moderate expressions in normal thymus and tumor‐adjacent tissues, and marginal expression in type A and type TSCC. The expression of TdT was also validated in our IHC analysis showing its strong positive signals in the type B thymomas, while only a marginal level was observed in the type A (Fig. 4B). On the other hand, CD99 was found with a different expression pattern showing a very high expression in type B, type AB, normal thymus, and adjacent tissues, as well as a moderate expression in type A and type TSCC tissues. Overall, the three specific proteins markers of immature T lymphocytes, namely TdT, CD1a, and CD99, were found at significantly higher level in type B thymoma than in type A (*P* < 0.05). In addition, the cortical epithelial cell markers such as PSMB11 (Beta5T), PRSS16, and Cathepsin V were also quantified in our study. PSMB11 displayed an increasing expression along with enhanced involvement of the type B thymoma component. Cathepsin V showed a very unique expression profile with its presence only in type B thymoma and slightly in adjacent tissues. Similar results were also observed in our IHC analysis (Fig. 4B). Interestingly, another cortical epithelial cell marker in thymoma (Marx *et al.*, 2014; Wu *et al.*, 2016), PRSS16, showed no significantly different expression between type B (cortical) and type A (medullary) in our proteomic survey (Fig. 4A). Similar cases were also observed on desmin (medullary) and panCK (Fig. S7). Overall, our DIA‐based proteomic survey presented consistent expression patterns for a major set of the TET subtype classifiers that are clinically practiced.

### 3.4 Validation of classifiers to differentiate type A and type B thymomas via parallel reaction monitoring and immunohistochemistry

**Figure 5:**
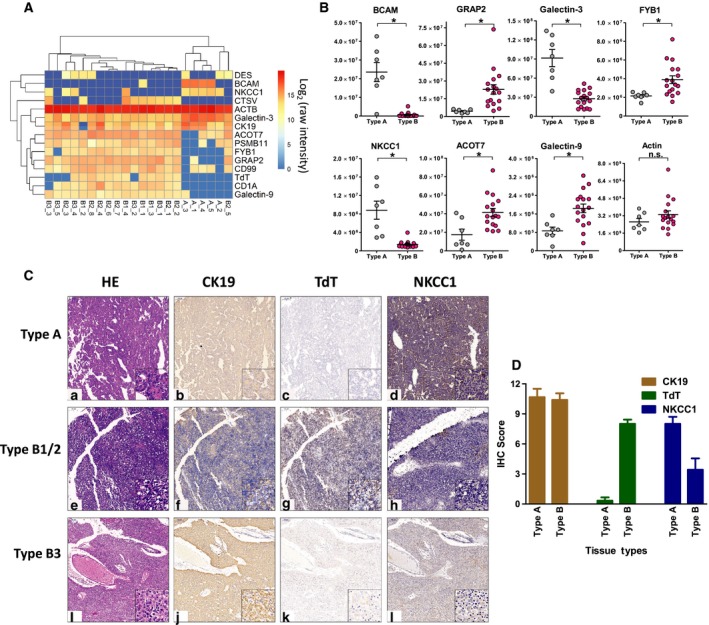
Validation of classifiers to differentiate type A and type B thymomas via PRM and IHC. (A) Representative differential protein markers identified in discovery experiments to distinguish type A (*n* = 5) and type B (*n* = 17) thymomas (with log_2_ protein abundance), in which ACOT7, BCAM, FYB1, galectin‐3/9, GRAP2, NKCC1, and actin (ACTB) were included in PRM target list. (B) PRM results of selected proteins. The quantitative difference (raw MS abundance) in target proteins between type A (*n* = 7) and type B (*n* = 17) thymomas retained significant, while no significant difference in actin abundance was observed between the two groups. Data were shown as mean ± SEM. Statistical significances were determined by Student’s *t*‐tests (an asterisk (*) indicated a significant change in protein abundance between the two groups (*P* < 0.05) and ‘n.s.’ represented ‘not significant’). (C) In situ IHC stainings of NKCC1 on thymoma samples together with CK19 (to localize thymoma epithelia) and TdT (to identify immature T lymphocytes which predominantly present in type B thymoma) were conducted, showing widely expression of NKCC1 in tumor epithelia of type A thymomas (main images were at 100× magnification, and zoomed images on lower right were at 400× magnification). (D) Semiquantitative view of marker expressions by scoring of the IHC images. Results were in line with proteomic studies: The scores of NKCC1 were lower in type B thymoma (*n* = 4) compared with type A (*n* = 7). Data were shown as mean ± SEM.

Mass spectrometry‐based targeted proteomic approaches were nowadays widely applied for biomarker studies due to its outstanding performance in terms of high sensitivity, reproducibility, and throughput (Gallien *et al.*, 2012; Kim *et al.*, 2015; Peterson *et al.*, 2012; Ronsein *et al.*, 2015). In this study, we used PRM technique to validate a series of protein markers prioritized from discovery proteomics (DIA‐MS) and probed their *in situ* expression patterns by IHC stainings. For this purpose, a new cohort of 44 patient samples with different TET subtypes was incorporated, with 34 samples utilized for PRM analysis and 28 for IHC analysis (18 samples were analyzed by both PRM and IHC). For type A and type B thymoma classification, seven protein classifier candidates identified in the previous discovery DIA experiments, including ACOT7, BCAM, FYB1, galectin‐3/9, GRAP2, and NKCC1, were selected in our PRM assay due to their significantly differential expression patterns between these two subtypes (Fig. 5A). The abundance of actin was monitored for cross‐sample as a control. The PRM results are summarized in Fig. 5B (for raw fragment abundances of all PRM samples, see Table S3). The quantitative difference in these classifier candidates between type A and type B thymomas retained significant, while no significant difference in actin abundance was observed between the two groups, indicating the excellent specificities and cross‐sample reproducibilities of these candidate markers (Fig. 5B). Further, we conducted *in situ* IHC stainings of NKCC1 on thymoma samples together with CK19 (to localize thymoma epithelia) and TdT (to identify immature T lymphocytes that predominantly present in type B1/B2 thymoma). Result showed that NKCC1 was widely expressed in type A thymoma tissues and mainly localized in tumor epithelial cells. The immunoreactivity of NKCC1 was stronger and more stable in type A thymomas, compared with type B (Fig. 5C). We applied a semiquantitative scoring of the IHC images based on both staining intensity and number of positive cells. The higher the score is, the more positive cells and higher intensity the sample was observed. Again, the scores of NKCC1 were lower in type B thymoma compared with type A, whereas the scores of TdT were much higher in type B thymomas as expected. The scores of CK19 between two groups were similar (Fig. 5D).

### 3.5 Differential proteome between thymoma and thymic carcinoma

TSCC had recently been recognized as a less common (< 10%) but very malignant lesion of all TETs. Compared with thymoma, TSCC exhibited obvious cell anaplasia and is a distinctive tumor associated with poor survival rate and high recurrence. However, it was also observed that many TSCC and thymoma (mainly B3) were often mixed, leading to a potential speculation that TSCC might originate from a preexisting thymoma. To evaluate this possibility, we compared protein profiles between thymoma and TSCC samples. In addition, we also aimed to uncover any potential classifiers with adequate selectivity and specificity toward early and accurate diagnosis and prognosis of TSCC.

**Figure 6:**
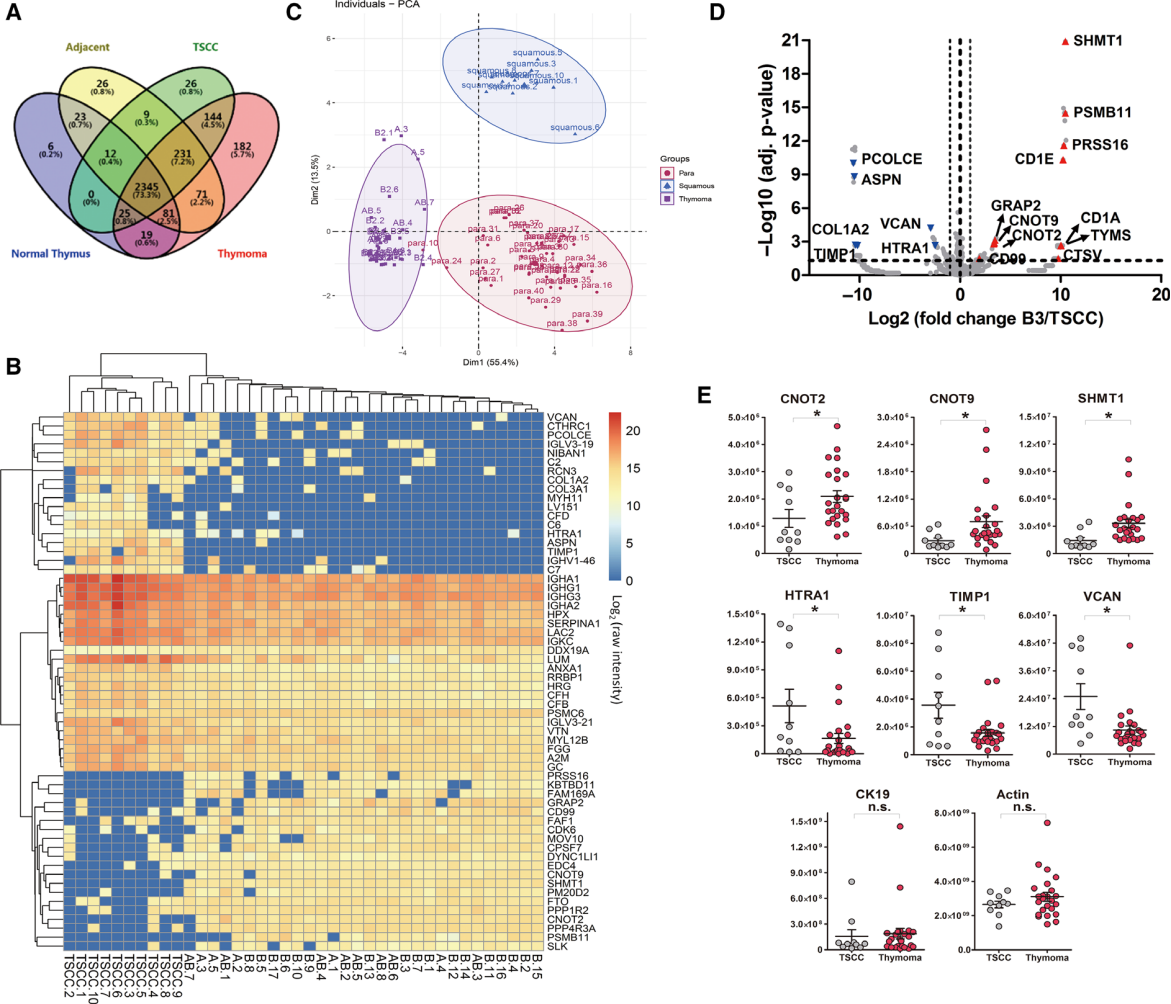
Differential proteome between thymoma and TC (TSCC). (A) Venn diagram of protein identifications among thymoma, TSCC, adjacent, and normal thymus tissues. (B) Hierarchical clustering analysis and heat map presentation of 60 differential proteins between thymoma and TSCC (in log2 protein intensity). (C) PCA using 60 differential proteins between thymoma and TSCC. Results demonstrated strong discriminative power of these proteins to differentiate TSCC from thymomas and the corresponding adjacent tissues as well (‘para’ represented ‘tumor‐adjacent tissue’, while ‘squamous’ stood for ‘TSCC’). (D) Volcano plot of B3 vs. TSCC. Volcano plot showed proteins such as SHMT1, CNOT2/9, PRSS16, and PSMB11 were found with increased expression in type B3 thymoma (*n* = 5), while proteins, for example, ASPN, VCAN, and HTRA1, were found less abundant in B3 than in TSCC (*n* = 10). Statistical significances were determined by Student’s *t*‐tests. (E) PRM validation of TSCC/thymoma markers such as CNOT2/9, SHMT1, VCAN, HTRA1, and TIMP1 in parallel with CK19 and actin (*y*‐axis showing raw MS abundance, an asterisk (*) indicated a significant change in protein abundance between the two groups, and ‘n.s.’ represented ‘not significant’). Statistical significances were determined by Student’s *t*‐tests. The expression of VCAN, HTRA1, and TIMP1 was significantly increased in TSCC samples (TSCC, *n* = 10; thymoma, *n* = 24), while CNOT2/9 and SHMT1 presented in TSCC samples with very low abundance. No significant differences in protein abundance of CK19 and actin were detected between thymoma and TSCC samples. Data were shown as mean ± SEM.

As shown in Fig. 6A, > 80% of total identified proteins were shared between thymoma and TSCC tissues. About 180 and 26 proteins are exclusively discovered in either thymoma or TSCC samples, respectively. Further significance test revealed 60 proteins that are differentially expressed between thymoma and TSCC (*P* < 0.05, Fig. 6B). Correlation analysis indicated that the expression profile of these proteins is highly correlated among samples within each group (thymoma or TSCC), but has much less correlations between these two groups (Fig. S8A). PCA of these proteins demonstrated that they have strong discriminative power to differentiate TSCC from thymomas (Fig. 6C) and the corresponding adjacent tissue as well. Such thymic proteins, with their capability of differentiating not only between thymoma and TSCC, but also between tumor and their adjacent tissues, have empowered them great potential as clinical biomarkers for more precise clinical diagnostics.

Further comparison of type B3 and TSCC proteome revealed a number of differential proteins with similar expression patterns: CNOT2/9 and SHMT1 were found with high abundance in type B3 thymoma, while the abundance of HTRA1, TIMP1, and VCAN was higher in TSCC than in type B3 thymoma (Figs 6D and Fig. S8B). This information has guided us to further validate these significant molecules on a new batch of patient samples. CNOT2, CNOT9, CD99, PRSS16, PSMB11, and SHMT1 were found with high abundance in thymoma and participated mainly in nucleic acid and amino acid metabolism. Considering the fact that TSCC is more malignant than thymomas with poor survival and high recurrence, we explored the possibility whether the expression of these markers could be associated with prognosis. We probed the expression of CNOT2, CNOT9, and SHMT1 in an online RNA‐seq database (http://www.kmplot.com) and generated Kaplan–Meier survival curves of TET patients (*n* = 119) associated with the differential RNA expression of CNOT2, CNOT9, and SHMT1. It was inspiring to find that the expression of CNOT family (CNOT2 and CNOT9) and SHMT1 was highly correlated with the prognosis of thymoma: Patients with lower expression of these genes at transcription level had poor relapse‐free survival (RFS) probability (Fig. S9). On the other hand, proteins including TIMP1, COL1A2, VCAN, PCOLCE, and HTRA1 were identified in TSCC with increased expressions, many of which mainly involved in immune response and matrix metalloproteinase activities.

Attributed to the excellent multiplexing capability of PRM technique, it was feasible to evaluate a considerable number of protein targets simultaneously within one MS injection. Based on the same PRM validation sample batch as described before, we also examined TSCC/thymoma marker candidates such as CNOT2/9, SHMT1, VCAN, HTRA1, and TIMP1 in parallel with CK19 in the PRM analysis (for raw fragment abundances of all PRM samples, see Table S3). As expected, results were highly consistent with that from the discovery proteomic, showing that expression of VCAN, HTRA1, and TIMP1 was significantly increased in TSCC samples, while CNOT2/9 and SHMT1 presented in TSCC samples with very low abundance (Fig. 6E). No significant differences in control proteins of CK19 and actin were detected between thymoma and TSCC samples.

### 3.6 Low expression of CNOT2/9 and SHMT1 in the epithelia of TSCC samples

**Figure 7:**
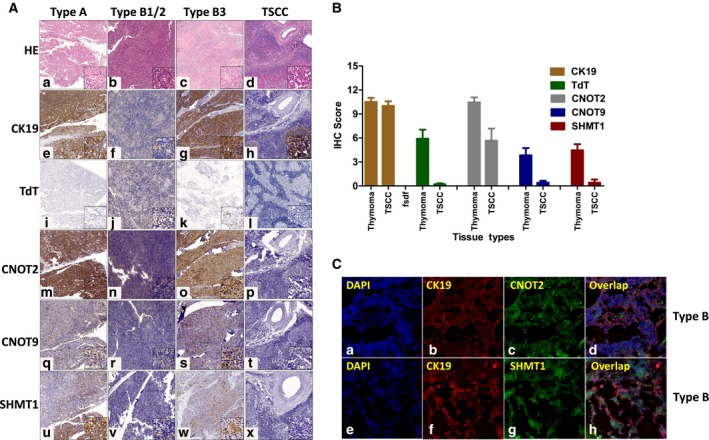
CNOT2/9 and SHMT1 levels were specifically low in the epithelia of TSCC samples. (A) Immunohistochemical stainings of CNOT2/9 and SHMT1 together with CK19 (KRT19) and TdT to index the location for tumor epithelial cells and immature T lymphocytes in type B thymoma, respectively. Main images were at 100× magnification, and zoomed images on lower right were at 400× magnification. CNOT2/9 and SHMT1 were found highly expressed in the epithelia in thymoma samples, but were barely in TSCC. (B) Semiquantitative scoring of all IHC images of CNOT2/9 and SHMT1 in thymoma and TSCC samples (sample numbers (thymoma/TSCC): CNOT2, 9/6; CNOT9, 12/5; SHMT1, 11/5). Data were shown as mean ± SEM. The scores for CNOT2/9 and SHMT1 were significantly lower than that in thymomas, which strongly validated our mass spectrometry results. (C) Immunofluorescent stainings of CNOT2 and SHMT1 by confocal microscopy showed their strong fluorescent signals in tumor epithelial cells indexed by CK19 (images were at 400× magnification).

In our targeted proteomic survey (DIA and PRM), CNOT2/9 and SHMT1 were found with differential expression patterns between thymoma and TSCC (Fig. S10 for DIA‐MS and Fig. 6E for PRM results), which were also found highly associated with prognosis. To further investigate whether these potential classifier proteins (namely CNOT2/9 and SHMT1) were mainly contributed by epithelial cells or lymphocytes in complex clinical tissue sections, we performed immunohistochemical stainings of CNOT2/9 and SHMT1 on the IHC validation sample batch as described earlier. CK19 (KRT19) and TdT were included in the IHC analysis to index the location for tumor epithelial cells and immature T lymphocytes in type B thymoma, respectively. As shown by representative IHC images in Fig. 7A, both CNOT2/9 and SHMT1 were overlapped with CK19, the marker of epithelial cells (Fig. 7A, images N, R, and V), but not with TdT, the marker of immature lymphocytes (Fig. 7A, image J), indicating that CNOT2/9 and SHMT1 were highly expressed in the epithelia in thymoma samples, but not in the epithelia of TSCC. These data suggested that the expression patterns of CNOT2/9 and SHMT1 in TETs were very likely associated with an epithelial mechanism of tumor/cancer cells. Furthermore, we conducted semiquantitative analysis of scoring on both intensity and number of positive cells of the IHC images. Consistently, the expression of CNOT2/9 and SHMT1 estimated by such semiquantitative scores in TSCC samples was significantly lower than that in thymomas (Fig. 7B, more images for TdT staining; see Fig. S11), which strongly validated our mass spectrometry results (Fig. 6E). To visualize their spatial localizations, we applied immunofluorescent stainings by confocal microscopy in type B thymoma tissues using fluorescent‐labeled antibodies of CNOT2, SHMT1, CK19 (to index thymomas epithelia), and DAPI (nuclei staining). Results showed CNOT2/SHMT1 exhibited strong fluorescent signal in the epithelial cells indexed by CK19 (Fig. 7C).

## 4 Discussion

Although rarely occurred worldwide, TETs showed extremely heterogeneous morphology and clinical outcomes. Its etiology and tumor biology remained to be further characterized. This study generated a comprehensive proteomic profiling resource data on 134 fresh‐frozen samples across different histology types, including normal thymus, and thymoma of subtypes A, AB, B1, B2, B3, and TSCC in together with their adjacent tissues. A proteome landscape of individual TET patient and nontumor thymus had thereof been delineated at a scale of several thousands of proteins, which was expected to complement the existing thymus genome and transcriptome data, providing the necessary base for multidimensional and integrated omic navigation of the thymic tumors. Taking advantage of the unique re‐mining feature of the DIA technology, the proteomic spectra data generated in this study could be further re‐mined in the future if new proteins of interest and their specific spectra become available. This is akin to a virtual library of tissue slides waiting for future IHC analysis when necessary protein targets and their antibodies become available.

Protein biomarkers applied in immunohistochemical analysis are important for accurate subtype determination for resected thymomas, particularly for those less prototypical ones. Although many protein markers had been reported and some of them (e.g., TdT, CD1a, CD99, PSMB11, Cathepsin V, and PRSS16) are routinely practiced in pathological laboratories, selection of these protein biomarkers varied significantly in different subtype classifications and in different pathological laboratories. Therefore, a systematic characterization of these markers across multiple clinical samples of all TET subtypes is of general interest but less likely being achieved through traditional IHC analysis due to its intrinsic low throughput and complicated experimental procedures. Owing to the excellent capability of multiplexing and reproducible quantification achieved by DIA‐MS in this study, we were able to evaluate the protein expression patterns of major diagnostic molecular markers across all major TET subtypes. Simultaneous examination of the markers in multiple TET subtype tissues could provide useful and critical information for clinical application. For example, although CD99 was found to have higher level in type B thymoma than in type A thymoma, it also showed high levels in normal thymus (Fig. 4A) as well as in a variety of other cell lines and tissues (Wilhelm *et al.*, 2014). Therefore, it was less likely to be used as specific biomarker in distinguishing type B thymoma. In clinical practice, better type B classifiers such as CD1a and TdT were preferred. PSMB11 (Beta5T) and Cathepsin V represented strong cortical epithelial cell markers for differentiating type A and type B thymoma, whereas PRSS16 showed almost no differentiating power between type A and type B. However, PRSS16 appears to be a strong classifier to differentiate the thymoma from TSCC and normal/adjacent thymus tissues.

Further comparison of protein expression profiles between thymoma and TSCC identified several extracellular matrix (ECM) proteins, such as CFD, TIMP1, and VCAN, that are overexpressed in TSCC and involved in complement activation and inflammation process. Complement system was traditionally believed to be associated with recognition of cancer cells and function as an effector to disrupt the cancer cells (Gros *et al.*, 2008; Kochanek *et al.*, 2018; Macor *et al.*, 2018; Potlukova and Kralikova, 2008; Rambach *et al.*, 2008; Reis *et al.*, 2018). However, many recent studies also reported its cancer‐promoting role through continuous inflammation in the tumor microenvironment featured by stromal gathering of growth factors, matrix remodeling proteases, cytokines, and chemokines (de Visser *et al.*, 2006). In addition, it was also reported that the complement activation could stimulate TGFB signaling (Bora *et al.*, 2005; Gionanlis *et al.*, 2008). Consequently, upregulation of these ECM proteins in TSCC suggests a concerted mechanism that undermined the tissue homeostasis and promotes tumorigenesis via a protumor microenvironment.

In this study, CNOT2/9 and SHMT1 were quantified in the discovery MS survey and successfully validated by both targeted MS and IHC analysis with high consistency (Figs 6E and 7A). CNOT2/9 showed significantly lower levels in the TSCC samples than those in the thymomas. This is consistent with the recent discovery by Faraji *et al.* (2014), showing that compared with normal breast tissue samples, expression of CNOT2 was much lower (16‐fold) in invasive breast carcinoma samples (*n* = 53). Furthermore, knockdown of CNOT on mice was shown to result in significant increase in pulmonary metastasis (Faraji *et al.*, 2014). All these data suggested potential inhibitory roles of CNOT protein in tumor metastasis. Decrease in their expression might thus lead to poor prognosis and high recurrence rates. Studies on mRNA‐seq data of thymoma confirm such notion by showing the correlation between the low levels of CNOT2/CNOT9 and the poor survival. However, in studies with other cancer types, CNOT2 was also reported to play roles in promoting tumor progression (Gupta *et al.*, 2017; Paone *et al.*, 2014; Sohn *et al.*, 2018), possibly by downregulation of MHC II expression as evidenced in several cell line and animal models (Rodriguez‐Gil *et al.*, 2017), therefore undermined immunosurveillance of cancer. Serine hydroxymethyltransferase 1 (SHMT1), a key enzyme catalyzing the folate‐dependent serine/glycine interconversion during cellular metabolism processes, showed an extremely low level of expression in the TSCC (Fig. 7A) and its indicated correlation with poor prognosis (Fig. S8). SHMT1 polymorphism was also shown to be correlated with clinical outcomes (Bahari *et al.*, 2016; Wang *et al.*, 2014).

Besides systematic evaluation of the existing subtype classifiers, special protein expression patterns also could gain us insights into their impacts on thymus functions and T‐cell maturation, particularly (a) proteins such as PRSS16 and PSMB11, which were closely related to thymus function and played pivotal roles in development of CD8‐positive T cell, showed in considerable quantity in thymoma samples of all major subtypes (compared with TSCC samples). This indicates that the normal function of thymus in T‐cell development, especially the active role of thymic epithelial cells, was still partially retained even in neoplastic thymic epithelial cells in thymoma; (b) it was reported that downregulation of either CNOT subunit resulted in elevated expression of major histocompatibility complex class II (MHC II) in several ‘loss‐of‐function’ cell lines and animal models (Rodriguez‐Gil *et al.*, 2017). Low expression of MHC II was featured in many thymomas, which has a strong impact on normal T‐cell development (Weksler and Lu, 2014). In this study, we found elevated expression of CNOT2/9 in thymic epithelial cells in thymoma samples (compared with tumor‐adjacent normal tissues), which might have contributed to poor expression of MHC II in thymomas, thereby possibly affected T‐cell receptor signaling pathways (Hennecke and Wiley, 2001), and led to dysregulated T‐cell development. The abnormally developed T cell might impair immunosurveillance of cancer and could also cause autoimmune diseases. This was well‐evidenced in clinic that autoimmune diseases such as myasthenia gravis (MG) were often associated with thymoma. However, further functional studies considering thymus abnormalities and its role in T‐cell maturation will be definitely desired on more ideal and available samples such as cultured cells.

Compared with conventional subtyping markers (e.g., TdT, PRSS16, and PSMB11), CNOT2/9 and SHMT1 not only exhibited good segregating capabilities for thymoma and TSCC, but also showed great prognosis indicating potentials. Standing alone or in combination with existing subtyping markers, the discovery of CNOT2/9 and SHMT1 will facilitate future biomarker development for TET diagnosis toward diversified options, higher specificity, and more prognosis information to note. Although detail mechanisms and specific roles of these proteins in tumor progression and metastasis remain to be further characterized in the future, identification of CNOT2/9 and SHMT1 as strong indicators for prognosis and progression in TETs represents a significant advance in discovering novel prognostic biomarker of the TETs by proteomic approaches.

## 5 Conclusions

Overall, we generated a resource of molecular profiling data and delineated a comprehensive proteome map from individual thymus TET/tissue samples. It is the first time that proteome signatures of up to 134 fresh‐frozen samples were generated, leading to the most comprehensive TET proteome map so far which extends the existing human tissue proteome atlas (Kim *et al.*, 2014; Uhlen *et al.*, 2015; Wang *et al.*, 2019; Wilhelm *et al.*, 2014) and provides a useful baseline map of protein expression in the thymus. A panel of clinically practiced diagnostic‐assisting markers was evaluated across all major TET subtypes in this study, providing systematic view of the subtyping marker specificities. Significantly, we identified several new marker proteins featuring subtype and prognosis potentials for TETs. In particular, we discovered and validated for the first time that protein CNOT2/9 and SHMT1, showed dramatic low expression in thymic epithelia of TSCC. Probing of CNOT2/9 expression in the TCGA database further revealed their close correlation with the prognosis of TET patients. Incorporation of such newly developed subtyping and prognosis markers is expected to expand current rational treatment options for precise TET care. Furthermore, permanent digital proteome map generated in this study by DIA‐MS allows future re‐analysis and re‐mining of data *in silico* as our knowledge and sample resource advances, which is extremely practical for precious and nonrenewable clinical specimen, to assist investigation of thymus functions and pathogenesis of TETs as well as the development of evidence‐based treatment and prognosis management for TETs in the future.

## Conflict of interest

The authors declare no conflict of interest.

## Author contributions

XK and QS executed the sample preparation and DIA‐MS experiments; XK and CM performed data analysis; QS, ZG, and NX collected the tissue samples and clinical information; LZ and YH executed pathological evaluation of all recruited samples; XY performed IHC experiments; and WY and WF conceptualized and supervised all experiments and wrote the paper. All authors approved the final manuscript.

## Supporting information


**Abbreviation S1.** Abbreviations.

**Fig. S1.** Typical organ‐like characteristics that can be observed in thymoma samples.
**Fig. S2.** Principal Component Analysis of all identified protein across all samples.
**Fig. S3.** Principal Component Analysis (PCA) of all identified protein in normal thymus and adjacent normal tissues.
**Fig. S4.** Proteomic profile of type A and type B thymomas.
**Fig. S5.** Differential proteome between thymoma type A and type B3.
**Fig. S6.** Examples of confusing cases of thymoma type AB in the sample cohort.
**Fig. S7.** The expression profile of desmin and panCK across all subtypes.
**Fig. S8.** Correlation analysis of the expression profile of 60 differential proteins among TET samples and Heat map analysis of the expression patterns of differential proteins between B3 and TSCC (type A was also included for comparison).
**Fig. S9.** The expressions of CNOT2/9 and SHMT1 were associated with prognosis in mRNA level.
**Fig. S10.** Heat map and clustering analysis for differential proteins between thymoma and TSCC (log2 protein intensities).
**Fig. S11.** Selected images of TdT staining on different tissue sections.

**Table S1.** Clinical information of all recruited patients.

**Table S2.** DIA‐MS quantification data matrix for all samples (log2 intensity).

**Table S3.** Raw abundance of target proteins for all PRM samples.

**Table S4.** Antibody information used in this study.

**Table S5.** 155 differentially expressed protein between Type A and Type B (all log2 raw intensities in DIA‐MS).
