# Supplementary material for: Deciphering tissue‐based proteome signatures revealed novel subtyping and prognostic markers for thymic epithelial tumors
Source: Mol Oncol. 2020 Feb 6;14(4):721–41. doi: 10.1002/1878-0261.12642 (PMC7138395; doi:10.1002/1878-0261.12642)
Supplement: Supplementary file 2 — Fig. S1. Typical organ‐like characteristics that can be observed in thymoma samples. Fig. S2. Principal Component Analysis of all identified protein across all samples. Fig. S3. Principal Component Analysis (PCA) of all identified protein in normal thymus and adjacent normal tissues. Fig. S4. Proteomic profile of type A and type B thymomas. Fig. S5. Differential proteome between thymoma type A and type B3. Fig. S6. Examples of confusing cases of thymoma type AB in the sample cohort. Fig. S7. The expression profile of desmin and panCK across all subtypes. Fig. S8. Correlation analysis of the expression profile of 60 differential proteins among TET samples and Heat map analysis of the expression patterns of differential proteins between B3 and TSCC (type A was also included for comparison). Fig. S9. The expressions of CNOT2/9 and SHMT1 were associated with prognosis in mRNA level. Fig. S10. Heat map and clustering analysis for differential proteins between thymoma and TSCC (log2 protein intensities). Fig. S11. Selected images of TdT staining on different tissue sections. [file MOL2-14-721-s002.pptx]

## Slide 1
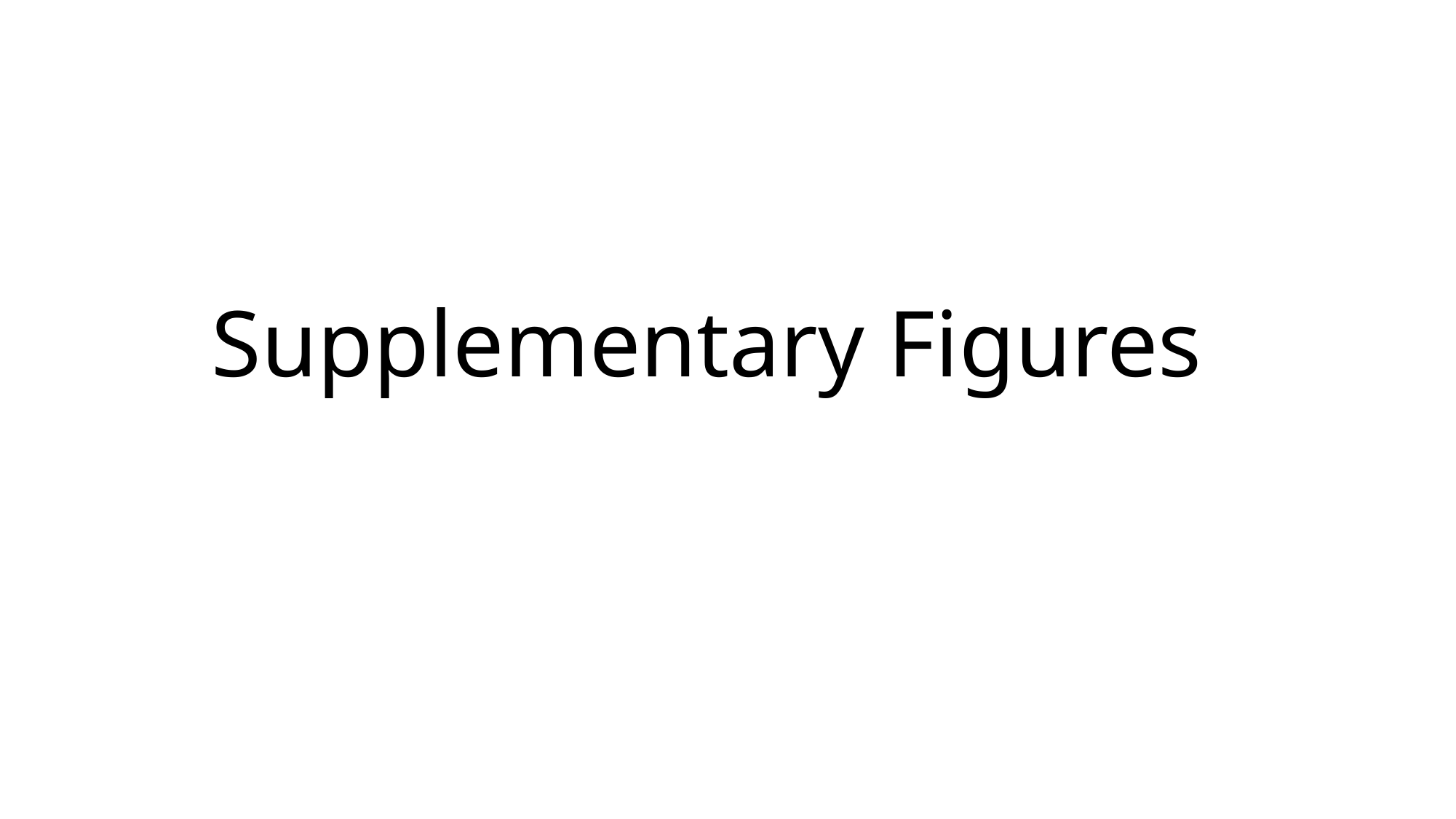

# Supplementary Figures

## Slide 2
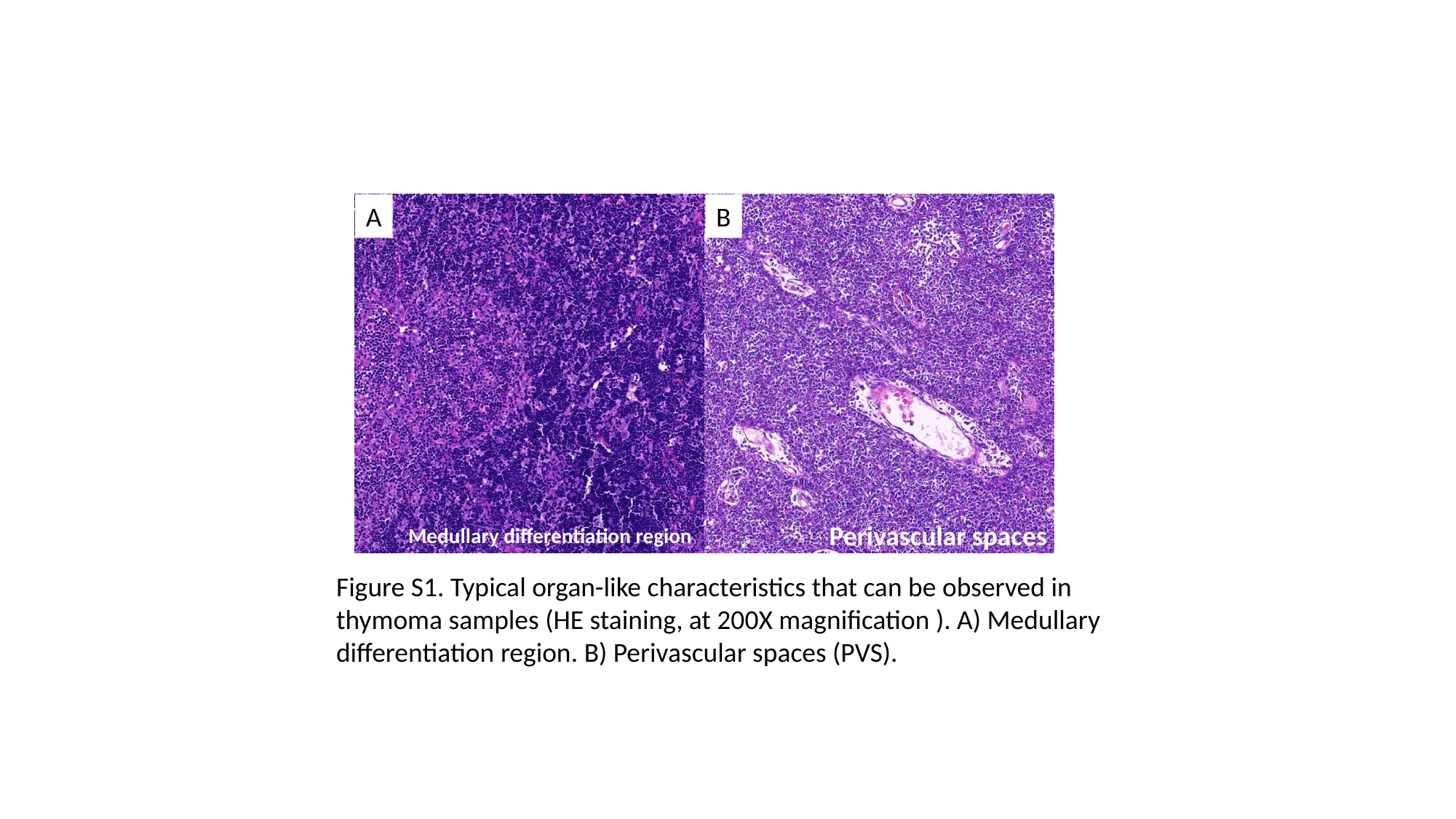

A
B
Perivascular spaces
Medullary differentiation region
Figure S1. Typical organ-like characteristics that can be observed in thymoma samples (HE staining, at 200X magnification ). A) Medullary differentiation region. B) Perivascular spaces (PVS).

## Slide 3
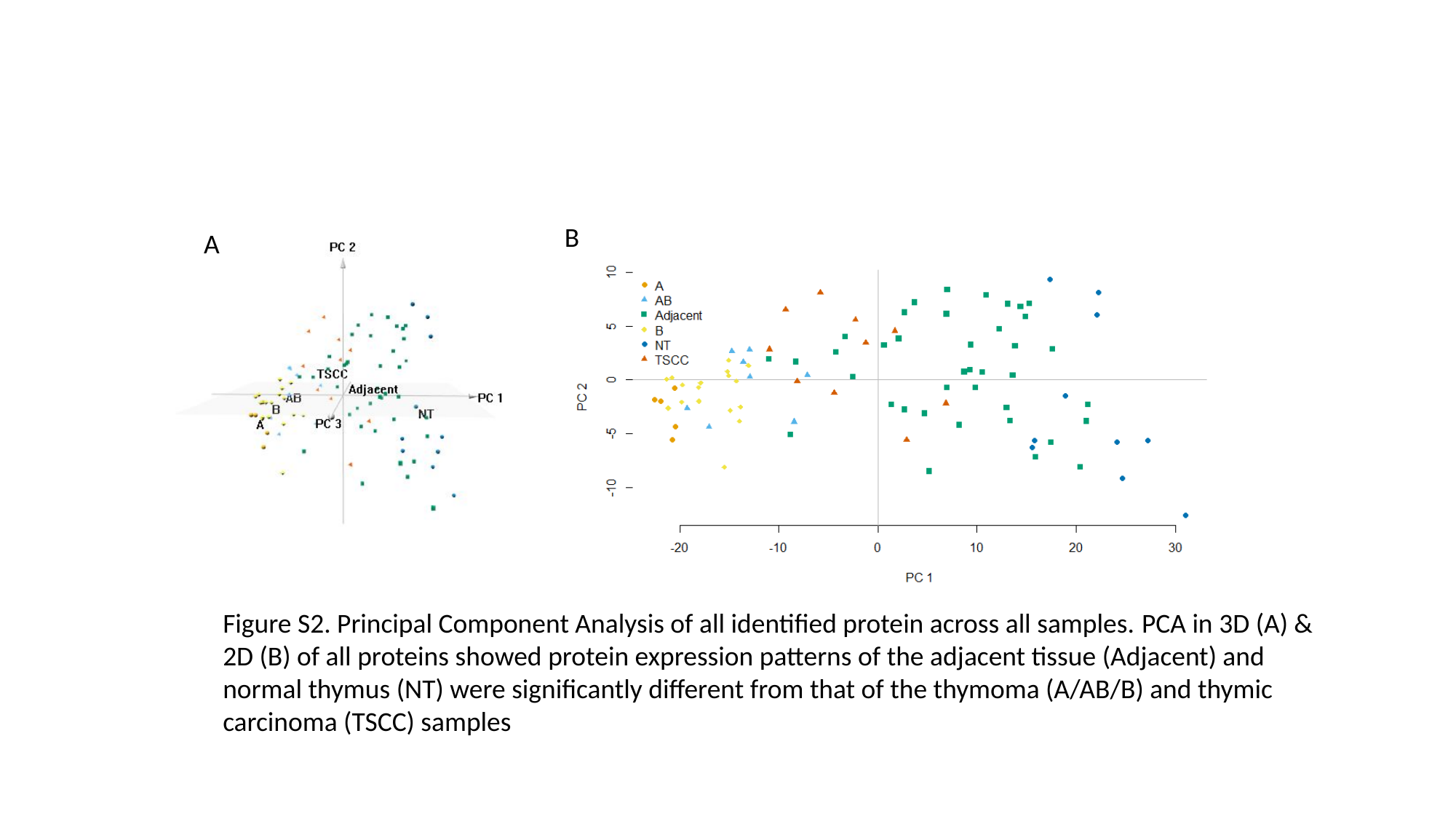

B
A
Figure S2. Principal Component Analysis of all identified protein across all samples. PCA in 3D (A) & 2D (B) of all proteins showed protein expression patterns of the adjacent tissue (Adjacent) and normal thymus (NT) were significantly different from that of the thymoma (A/AB/B) and thymic carcinoma (TSCC) samples

## Slide 4
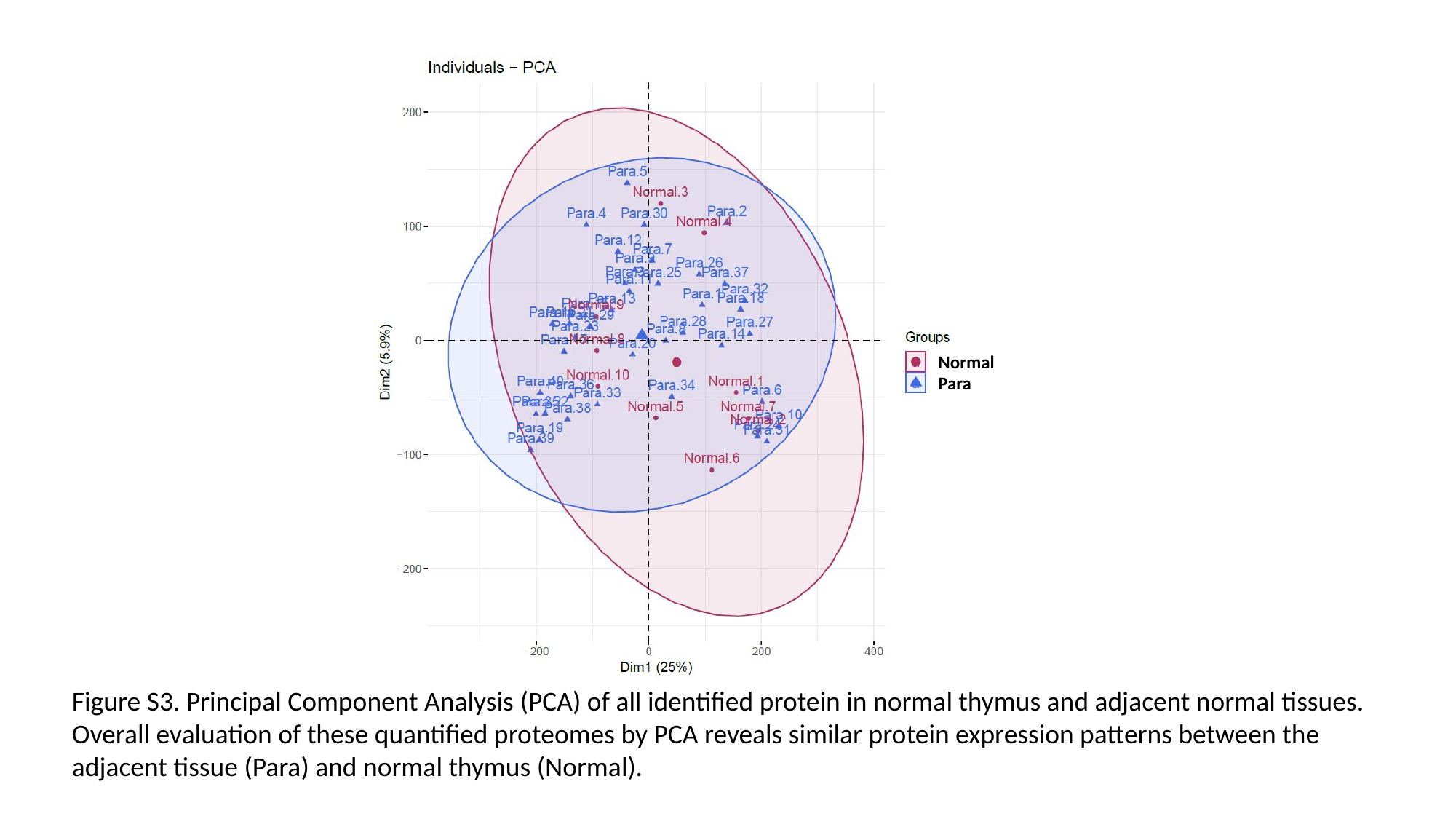

Normal
Para
Figure S3. Principal Component Analysis (PCA) of all identified protein in normal thymus and adjacent normal tissues. Overall evaluation of these quantified proteomes by PCA reveals similar protein expression patterns between the adjacent tissue (Para) and normal thymus (Normal).

## Slide 5
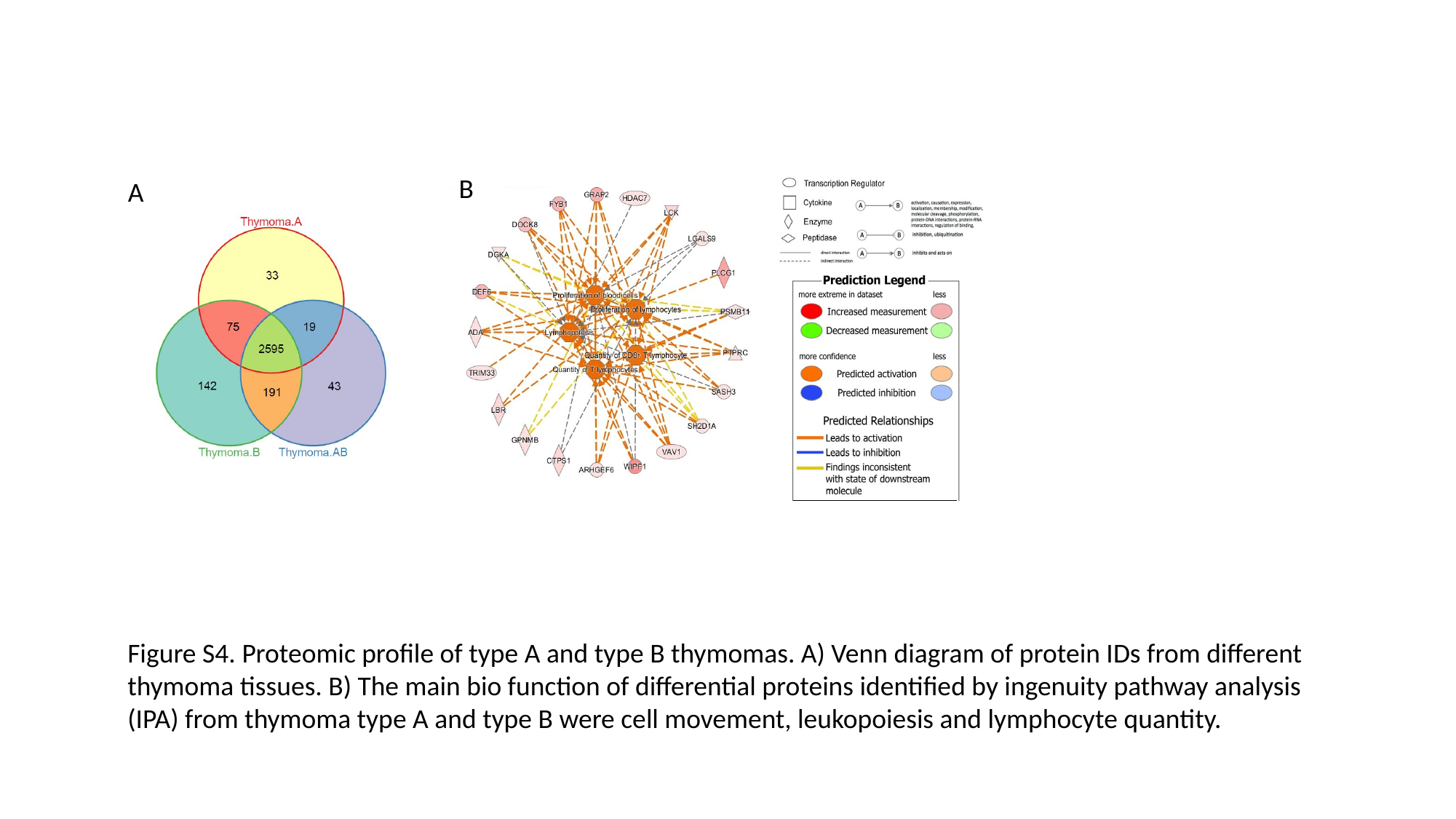

B
A
Figure S4. Proteomic profile of type A and type B thymomas. A) Venn diagram of protein IDs from different thymoma tissues. B) The main bio function of differential proteins identified by ingenuity pathway analysis (IPA) from thymoma type A and type B were cell movement, leukopoiesis and lymphocyte quantity.

## Slide 6
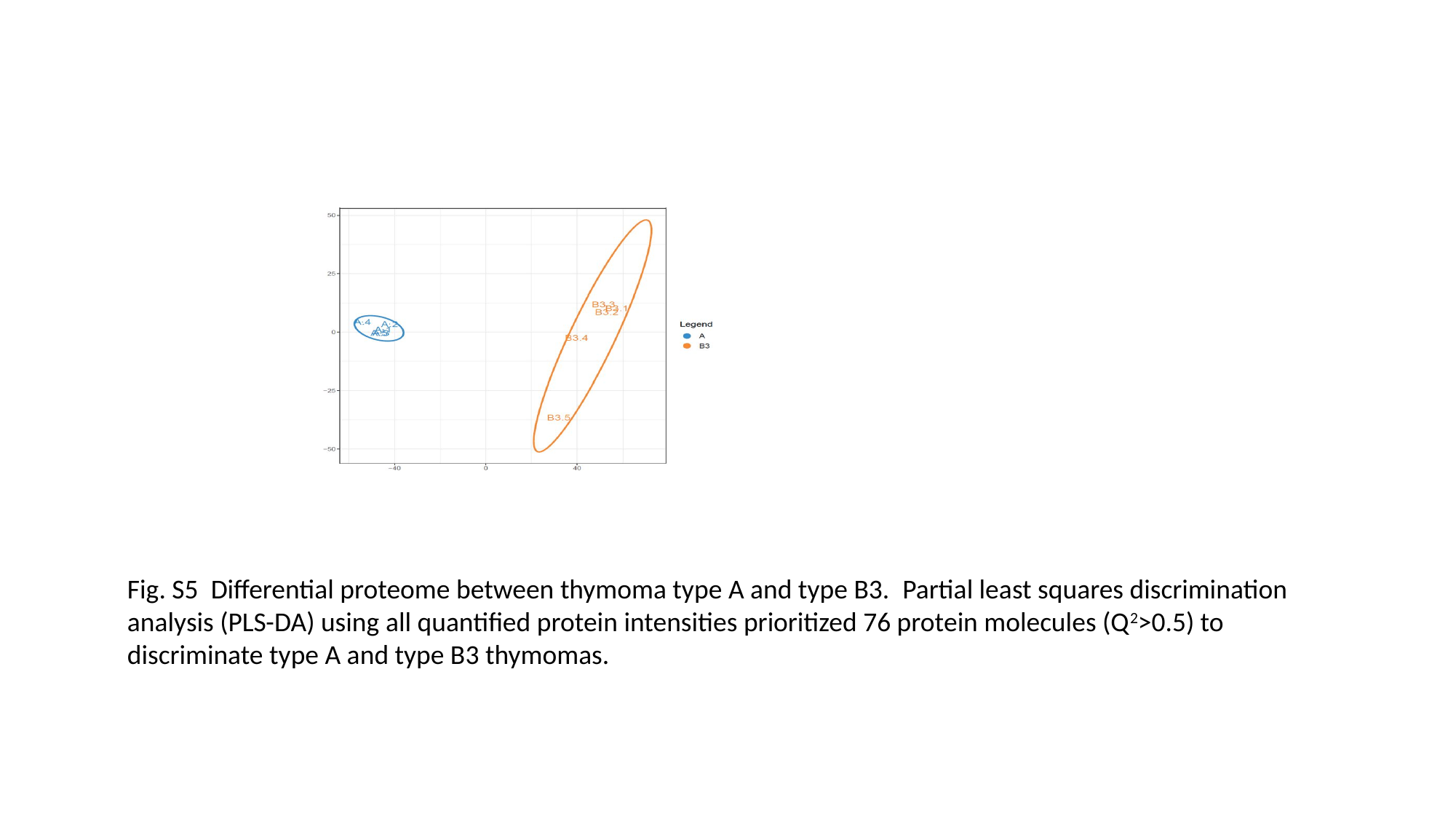

Fig. S5 Differential proteome between thymoma type A and type B3. Partial least squares discrimination analysis (PLS-DA) using all quantified protein intensities prioritized 76 protein molecules (Q2>0.5) to discriminate type A and type B3 thymomas.

## Slide 7
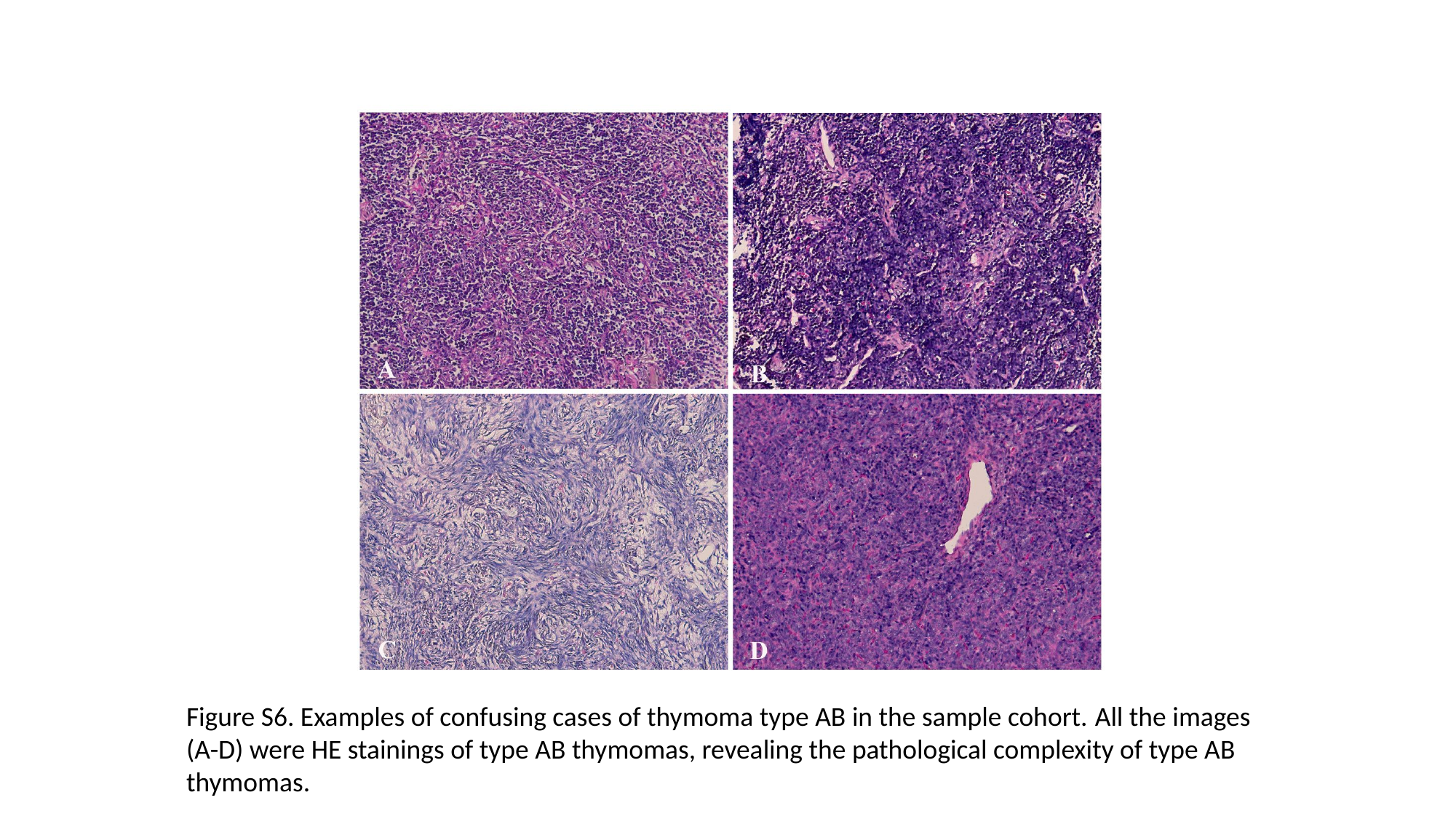

Figure S6. Examples of confusing cases of thymoma type AB in the sample cohort. All the images (A-D) were HE stainings of type AB thymomas, revealing the pathological complexity of type AB thymomas.

## Slide 8
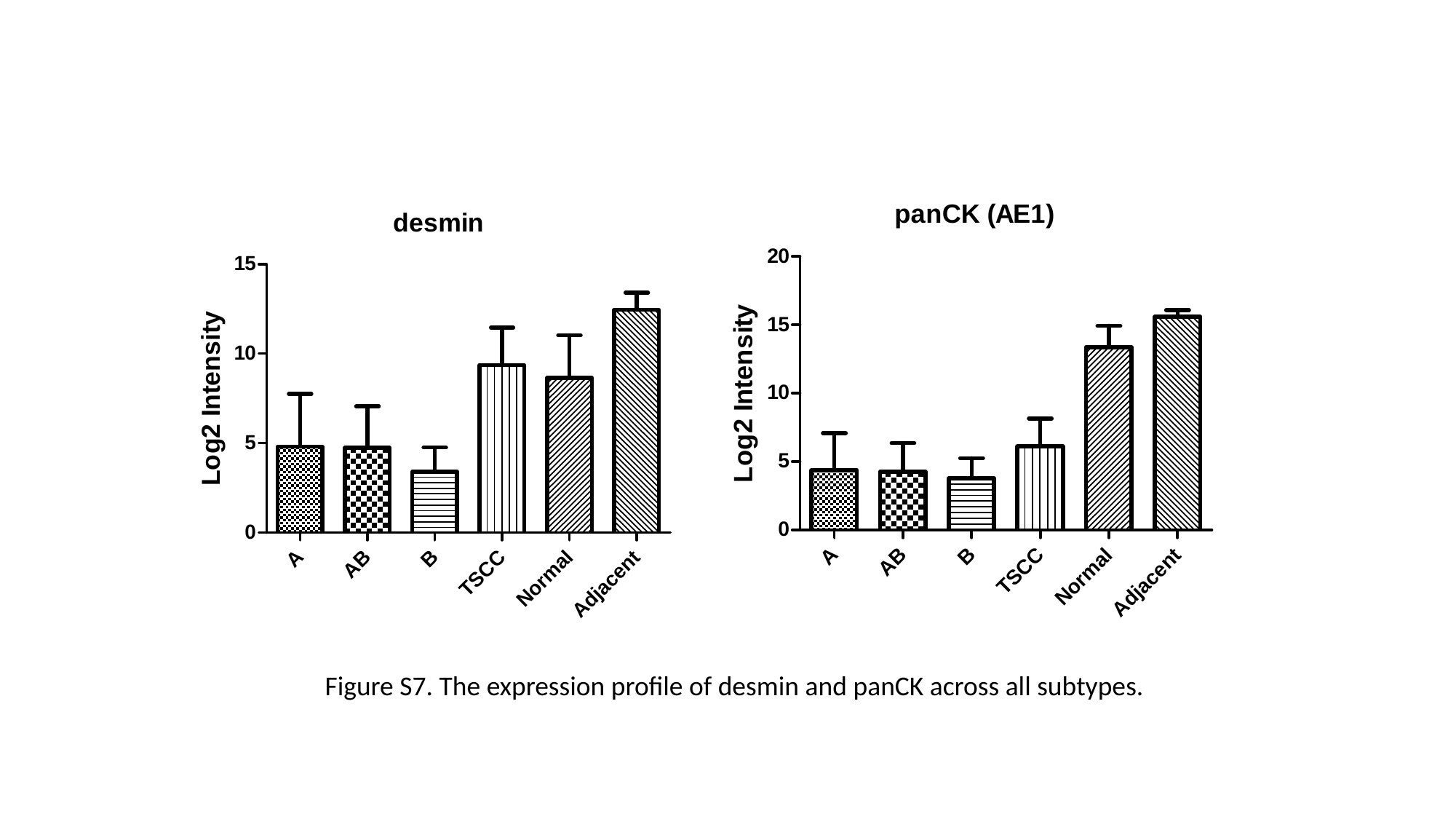

Figure S7. The expression profile of desmin and panCK across all subtypes.

## Slide 9
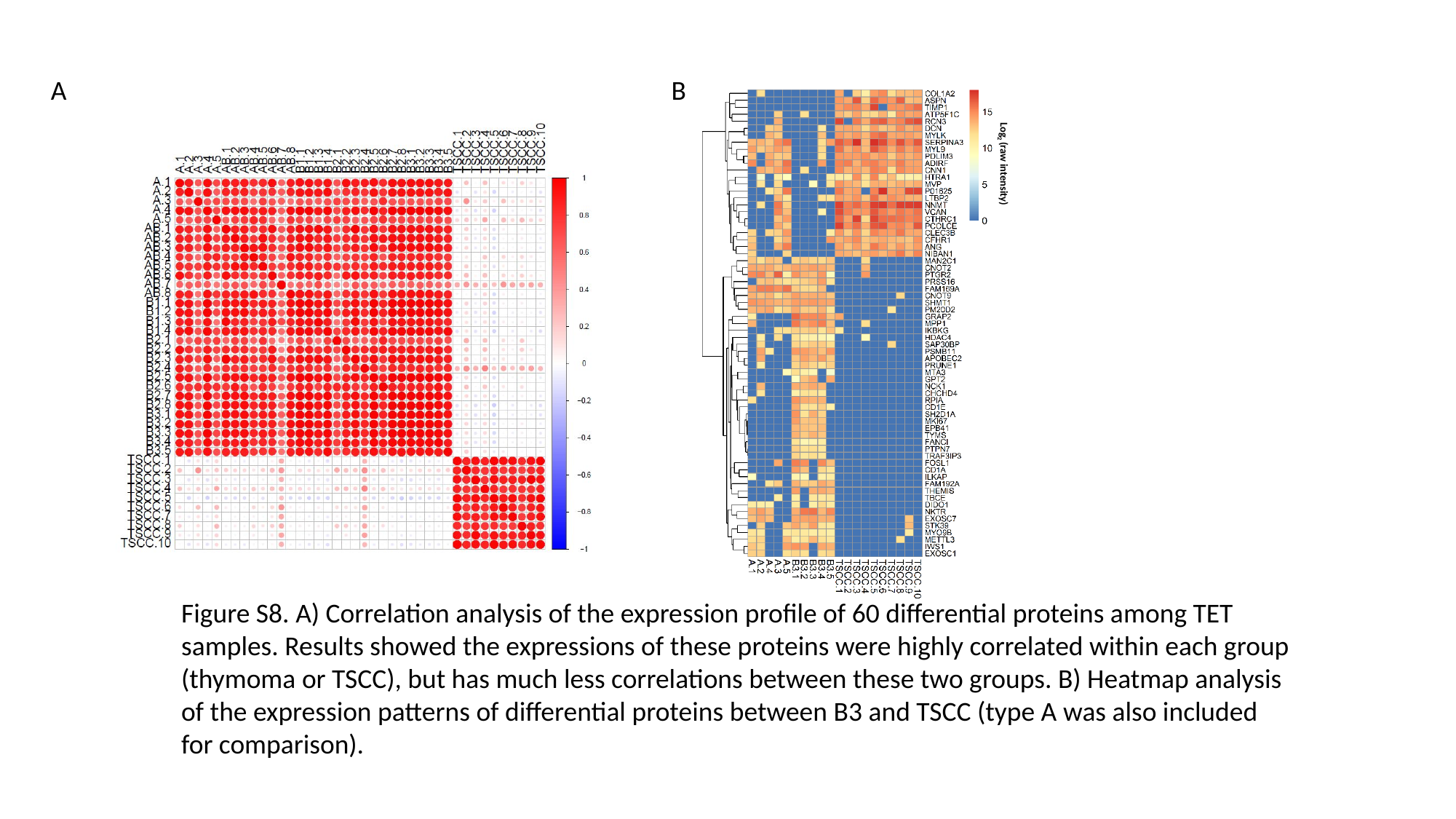

A
B
Log2 (raw intensity)
Figure S8. A) Correlation analysis of the expression profile of 60 differential proteins among TET samples. Results showed the expressions of these proteins were highly correlated within each group (thymoma or TSCC), but has much less correlations between these two groups. B) Heatmap analysis of the expression patterns of differential proteins between B3 and TSCC (type A was also included for comparison).

## Slide 10
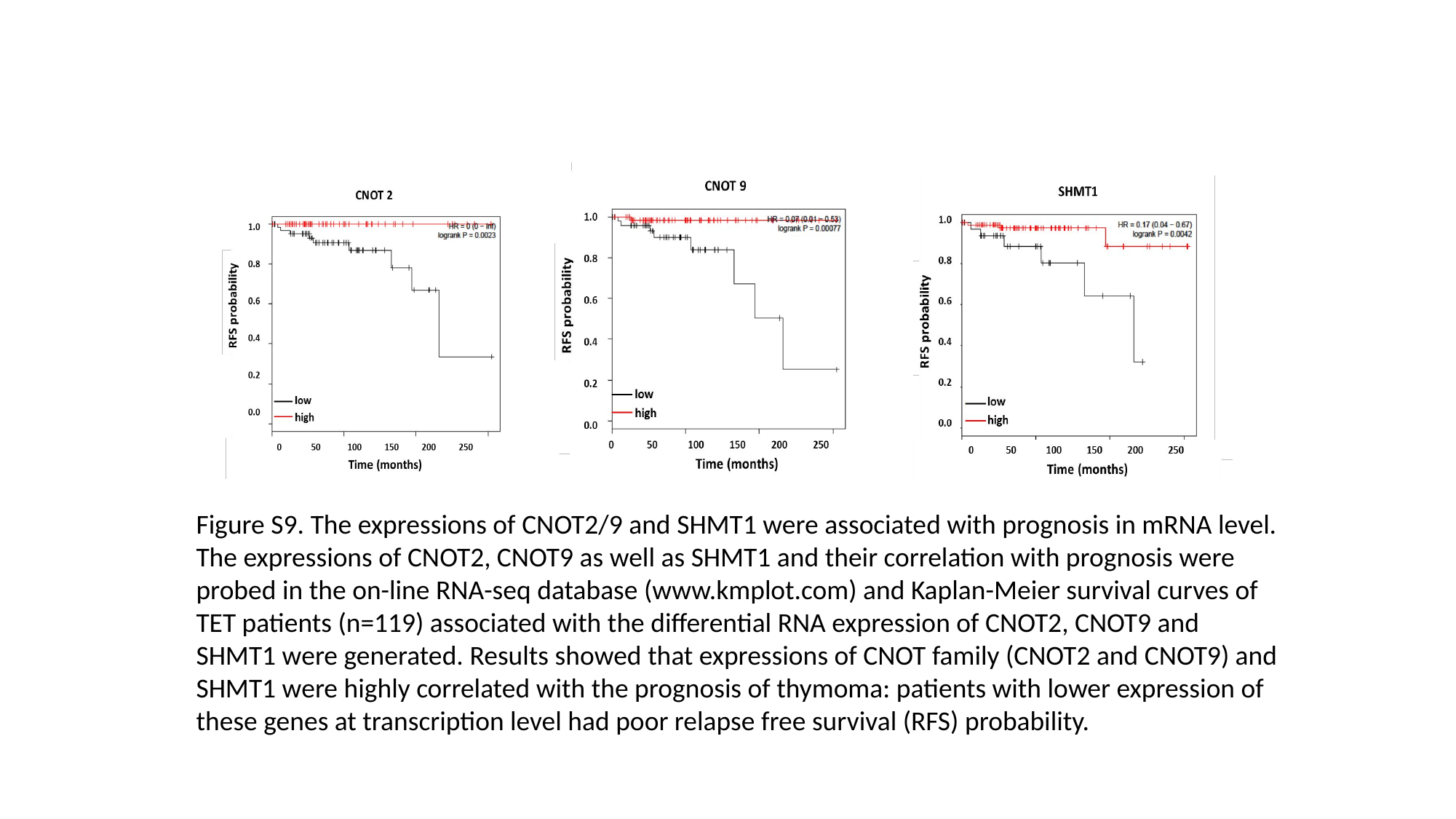

Figure S9. The expressions of CNOT2/9 and SHMT1 were associated with prognosis in mRNA level. The expressions of CNOT2, CNOT9 as well as SHMT1 and their correlation with prognosis were probed in the on-line RNA-seq database (www.kmplot.com) and Kaplan-Meier survival curves of TET patients (n=119) associated with the differential RNA expression of CNOT2, CNOT9 and SHMT1 were generated. Results showed that expressions of CNOT family (CNOT2 and CNOT9) and SHMT1 were highly correlated with the prognosis of thymoma: patients with lower expression of these genes at transcription level had poor relapse free survival (RFS) probability.

## Slide 11
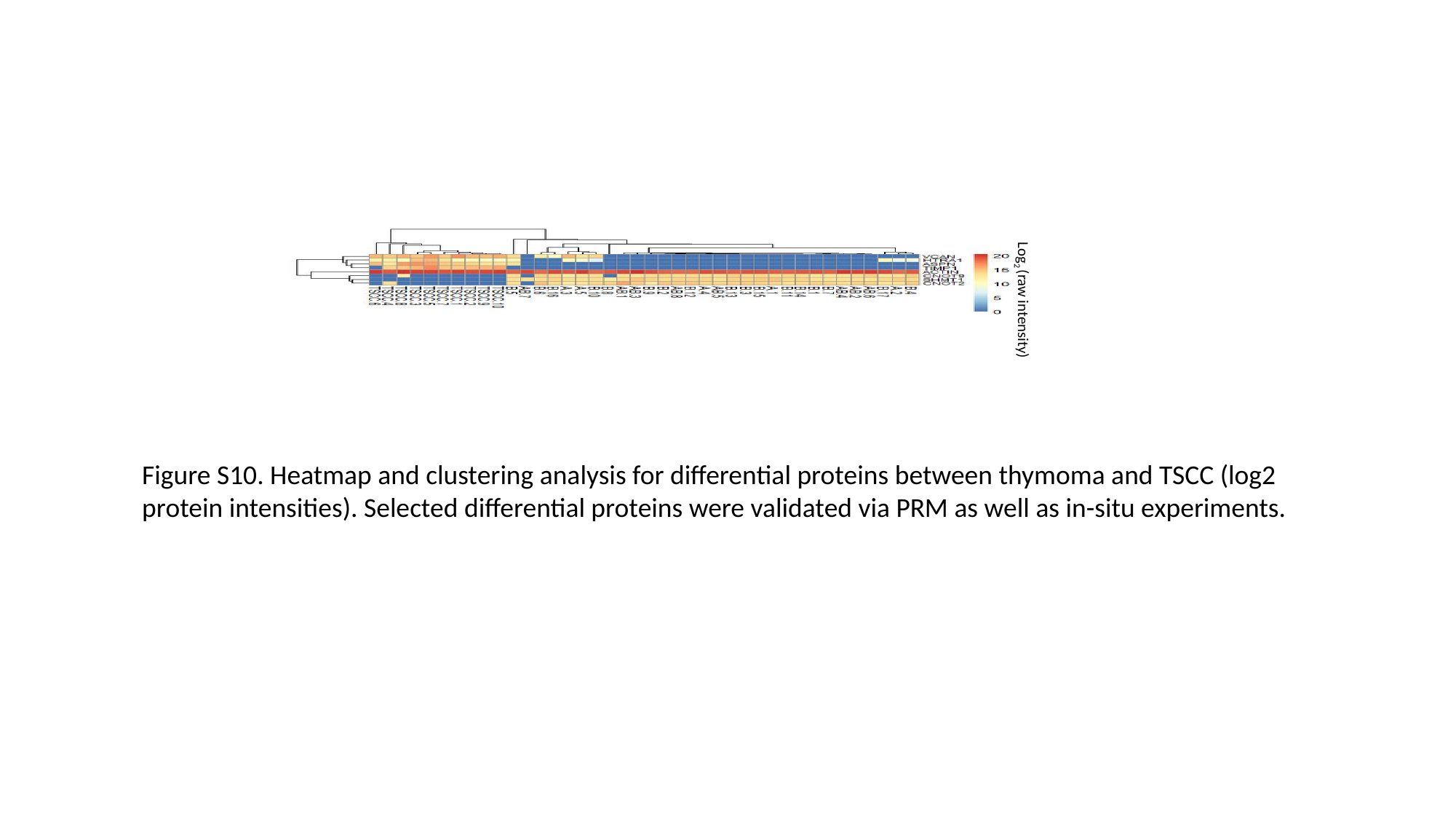

Log2 (raw intensity)
Figure S10. Heatmap and clustering analysis for differential proteins between thymoma and TSCC (log2 protein intensities). Selected differential proteins were validated via PRM as well as in-situ experiments.

## Slide 12
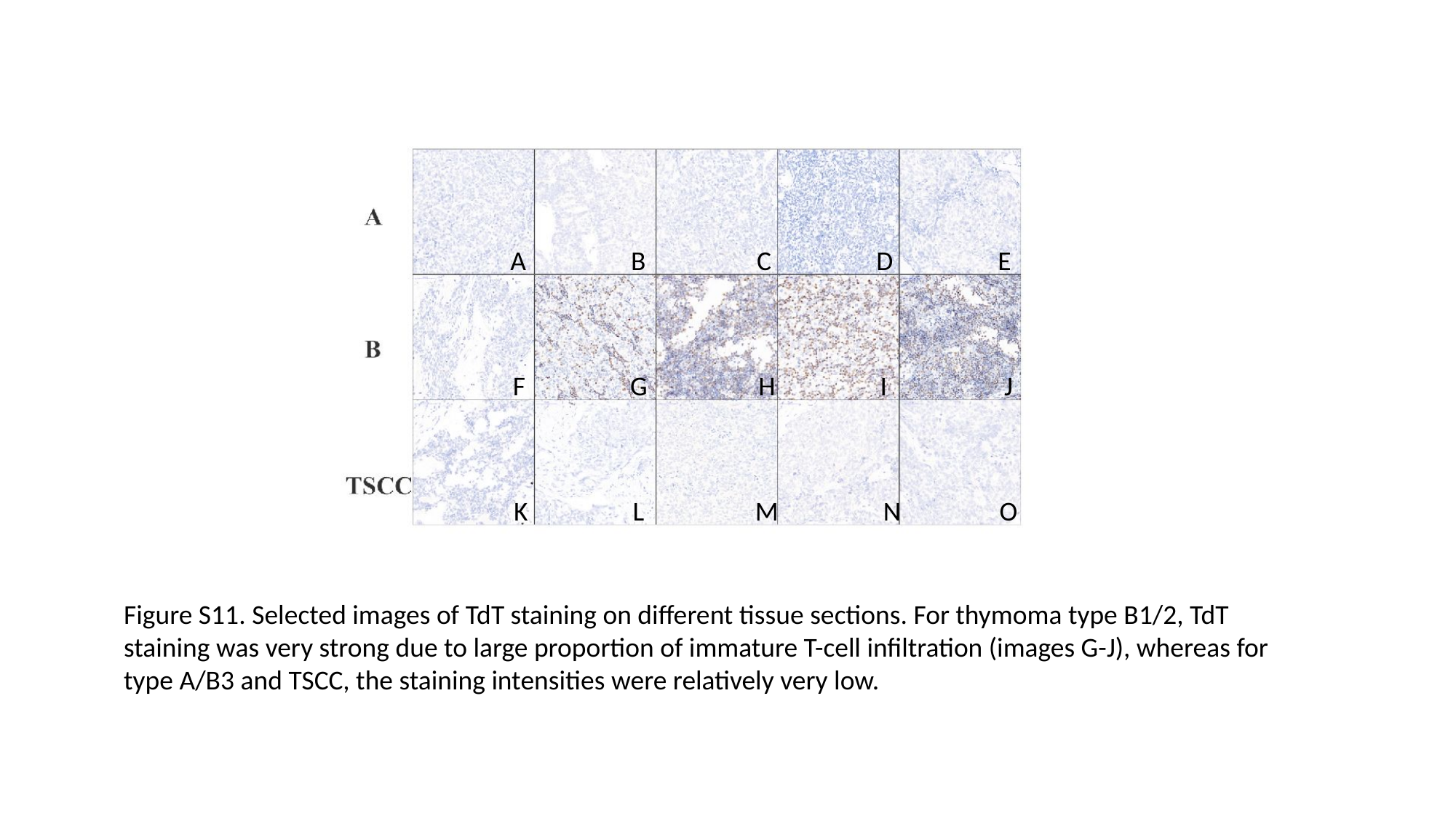

A B C D E
F G H I J
K L M N O
Figure S11. Selected images of TdT staining on different tissue sections. For thymoma type B1/2, TdT staining was very strong due to large proportion of immature T-cell infiltration (images G-J), whereas for type A/B3 and TSCC, the staining intensities were relatively very low.
